# Optimized exercise prescription for alleviating cancer-related fatigue in breast cancer survivors after primary treatment: a systematic review and dose-response network meta-analysis

**DOI:** 10.3389/fpubh.2026.1795975

**Published:** 2026-05-15

**Authors:** Zhonglin Dai, Yifan Zhang, Zhibo Wang, Xueying Chen, Peng Gao

**Affiliations:** 1 Graduate School of Physical Education, Myongji University, Yongin, Republic of Korea; 2 Shandong Vocational University of Foreign Affairs, Weihai, China

**Keywords:** breast cancer, cancer-related fatigue, exercise, meta-analysis, systematic review

## Abstract

**Background:**

Cancer-related fatigue (CRF) significantly impairs the quality of life in breast cancer survivors. Although exercise is recommended in clinical guidelines to alleviate CRF, the effective and optimal doses remain uncertain. This study aims to examine the dose–response relationship of different exercise modalities with CRF in breast cancer survivors.

**Methods:**

Exercise dose was calculated as the product of duration, frequency, and intensity, expressed in metabolic equivalents of task minutes per week (METs-min/week), for comparison across studies. Bayesian network and dose–response meta-analyses were conducted to evaluate the impact of exercise on fatigue in breast cancer survivors and to model the nonlinear dose–response relationship. The certainty of the evidence was evaluated using the Confidence in Network Meta-Analysis (CINeMA) web application.

**Results:**

A total of 32 studies involving 2,067 patients were included. An inverted U-shaped dose–response relationship between overall exercise and CRF was observed; a minimum effective threshold could not be definitively established, with a peak effect at 730 METs-min/week (SMD = 1.32, 95% CrI: 0.78, 1.89), and effects were no longer significant beyond 1,100 METs-min/week. Meta-regression analyses indicated that baseline fatigue severity significantly moderated exercise effects. No other covariates showed significant moderation. Subgroup analyses suggested that interventions lasting less than 12 weeks were more effective. When stratified by modality, aerobic exercise (0–830 METs-min/week; very low certainty evidence), combined aerobic and resistance training (0–1,200 METs-min/week; low certainty evidence), resistance training (0–450 METs-min/week; low certainty evidence) and yoga (0–500 METs-min/week; low certainty evidence) all showed significant associations with CRF reduction.

**Conclusions:**

This study provides an evidence-based optimized range of exercise doses across different exercise types for alleviating CRF in breast cancer survivors. Considering the low and very low certainty of the evidence, further high-quality studies are needed to confirm these findings and refine clinical decision-making.

**Systematic review registration:**

https://www.crd.york.ac.uk/PROSPERO/view/CRD420251146666, identifier: CRD420251146666.

## 1 Introduction

Breast cancer is the most common cancer among women, with nearly 2.3 million new cases diagnosed in 2022 (1). According to a global cancer statistics survey across 185 countries, it had the highest incidence in 159 countries (1). Despite steady improvements in survival rates worldwide (2), many survivors continue to face severe side effects that significantly impact their quality of life. This is primarily due to the substantial sequelae that breast cancer patients may endure following both the diagnosis and cancer-directed treatments (3).

Cancer-related fatigue (CRF) is a major and persistent side effect experienced by breast cancer patients (4). It encompasses physical, emotional, and/or cognitive exhaustion that is directly linked to cancer and its treatments (4). Unlike general fatigue, CRF is unpredictable, persistent, and cannot be relieved by rest or sleep (5). Approximately 43% of breast cancer survivors continue to experience CRF even after treatment, resulting in long-term impairments in quality of life (6). Given the complex causes and mechanisms of CRF, current guidelines do not offer specific recommendations for pharmacological treatments, underscoring the need for complementary and alternative therapies (5, 7).

Due to its well-established overall health benefits, exercise has been widely recommended as a supportive strategy for breast cancer survivors (8). The National Comprehensive Cancer Network's Clinical Practice Guidelines for CRF emphasize incorporating exercise as an adjunctive therapy in its management (7). Existing meta-analyses have primarily focused on the effects of exercise in alleviating fatigue or comparing the effects of different exercise modalities (9–16), with relatively little attention paid to the dose–response relationship. Given the unique physiological relationship between exercise and fatigue—where improvements in fatigue do not necessarily increase proportionally with higher exercise doses—it is essential to identify appropriate exercise doses specifically for CRF (17). In recent years, exercise oncology has increasingly moved toward individualized prescriptions, aiming to avoid a “one-size-fits-all” approach that merges different cancer types or treatment stages (18, 19). This shift highlights the need for more precise and high-quality evidence, particularly for breast cancer survivors who have completed primary treatment. Although subgroup analyses or meta-regression can be used to explore the moderating effects of exercise dose and intensity, these approaches rely on dichotomized cutoffs, which may increase the risk of biased results. For example, using 150 min per week as a cutoff would classify 60 min and 120 min of exercise into the same category, despite a twofold difference in actual dose. Furthermore, meta-regression is limited in its ability to characterize dose–effect relationships across the full dose spectrum. Pairwise meta-analyses also often cannot incorporate direct comparisons between different exercise doses, as they typically contrast exercise interventions only with no-exercise controls. Therefore, more precise dose assessment methods capable of incorporating the full spectrum of available evidence are needed to model potential nonlinear dose–response relationships. To fill this gap, a Bayesian framework for dose-response network meta-analysis would be recommended, as this method comprehensively accounts for the heterogeneity across various exercise types and doses (20). It has two distinct advantages: (1) it can determine the effective and optimal doses for various exercise modalities, and (2) unlike traditional network meta-analysis, which mainly identifies the best intervention, this approach provides a comprehensive ranking of different doses across modalities, allowing for a more precise determination of clinical applicability.

In summary, this study aims to map dose–response relationships between different exercise modalities and CRF in breast cancer survivors after primary treatment. Meta-regression will assess whether age, baseline fatigue, and intervention length moderate these relationships, as they are key sources of heterogeneity in exercise interventions. Subgroup analyses by baseline fatigue and intervention length will further explore response variability across patient and intervention characteristics. The findings will provide exploratory dose–response evidence to inform the development of optimized exercise prescriptions for post-treatment CRF.

## 2 Methods

### 2.1 Protocol and registration

This systematic review and network meta-analysis was pre-registered (PROSPERO registration number: CRD420251146666) and conducted in accordance with the PRISMA-NMA reporting guidelines (Supplementary Content 1) (21).

### 2.2 Search strategy

We searched several electronic databases, including PubMed, Web of Science, Embase, and the Cochrane Central Register of Controlled Trials, from their inception to November 25, 2025, with no language restrictions. The search incorporated keywords and MeSH terms related to the participants (breast cancer), intervention (exercise), outcome (fatigue), and study design (RCTs), combined using the logical operator AND. To identify additional eligible studies, we also manually searched the reference lists of relevant articles not indexed in the databases. A detailed description of the search methods for each database is provided in Supplementary Content 2.

### 2.3 Eligibility criteria

Eligibility criteria were established using the PICOS format (Population, Intervention, Comparison, Outcomes, and Study Design) to minimize clinical bias in study selection (22). Table 1 summarizes the eligibility criteria for included studies based on the PICOS framework.

**Table 1:** Eligibility criteria formulated based on the PICOS framework.

PICOS	Inclusion criteria	Exclusion criteria
Population	Adult women who are breast cancer survivors, defined as individuals who have completed primary treatment, including surgery, chemotherapy, and radiation therapy	Combining breast cancer with other cancers unless separate data for breast cancer patients were provided
Intervention	Intervention encompassed any form of exercise (i.e., structured, repeated physical activity aimed at improving or maintaining fitness)	Relaxation training; studies combining exercise with other interventions (e.g., nutritional supplementation), unless the control group received the same non-exercise components, allowing for accurate assessment of exercise effects.
Comparison	A control group (e.g., usual care) was required. For direct comparison studies, the control group could include any form of exercise intervention, including different intensities within the same exercise modality	Other interventions beyond exercise or usual care
Outcomes	Cancer-related fatigue was assessed using validated scales, such as the Brief Fatigue Inventory or the Functional Assessment of Cancer Therapy—Fatigue	Studies not assessing cancer-related fatigue
Study design	Only randomized controlled trials were included; for studies reporting the same trial, only the one with the largest sample size was included.	Non- randomized controlled trials, such as quasi-experimental and observational studies

### 2.4 Data extraction and coding

Two reviewers (DZL and ZYF) independently extracted data using a pre-designed Excel form, including study details (e.g., author/year, study region), participant characteristics (e.g., age, sample size), intervention specifics (e.g., exercise type, frequency, duration, and intervention length), control interventions (e.g., type, sample size), and outcomes (e.g., assessment tools, main results). Means and standard deviations (SDs) of CRF were recorded for both exercise and control groups at baseline and post-intervention. Any discrepancies were resolved through discussion or consultation with a third reviewer (GP). To estimate SDs of change scores between pre- and post-intervention, a correlation coefficient of 0.5 was used, reflecting typical measurement reliability (23). Missing SDs were derived from confidence intervals, *p*-values, or *t*-values; if data were unavailable, the original authors were contacted. Since dose-response relationships require the use of standard errors (SE) for the analysis, we converted SDs to standard errors using the following formula:


$$\begin{array}{l} \text{SE}=\frac{\text{SD}}{\sqrt{N}} \end{array}$$ (1)

where *N* represents the sample size.

The data were cross-checked for accuracy by the reviewers, and a third author (GP) verified the final dataset.

To conduct the dose-response network meta-analysis, we categorized interventions into three levels. Initially, interventions were classified as “Exercise” or “Control.” In the second level, exercise interventions were further categorized based on the type of exercise reported in the original studies: “Aerobic Exercise” (e.g., running, cycling, walking, dancing; AE), “Resistance Training” (e.g., resistance bands, weight training; RT), “Combined Aerobic and Resistance Training” (AE-RT), and “Yoga”. Table 2 presents detailed definitions of the different exercise modalities (24). At the third level, interventions were classified based on the total weekly exercise dose, calculated as the product of duration, frequency, and intensity, expressed in metabolic equivalents of task minutes per week (METs-min/week). For duration, warm-up and cool-down periods were excluded from the total exercise time. If exercise duration increased progressively over several weeks, the average total exercise time was used. For intensity, we referred to the 2024 Adult Physical Activity Guidelines and selected appropriate coding based on the exercise type and intensity (e.g., percentage heart rate max, rating of perceived exertion) reported in the original studies (25). This guideline provides METs values covering a wide range of exercise types, which were sufficient for allocating intensity in our analysis. AE intensity was coded according to exercise type and target percentage of maximum heart rate (%HRmax). RT intensity followed ACSM guidelines based on one-repetition maximum (%1RM; light < 50%, moderate 50%−69%, vigorous ≥70%−85%) (24); if %1RM was unavailable, intensity was inferred from RPE (≈12–13 = moderate; 14–17 = vigorous) or repetition ranges (12–20, 8–12, 6–8 reps). When studies reported only qualitative intensity levels, such as “moderate,” we applied the ACSM absolute-intensity thresholds (light ≈ 2.0–2.9 METs, moderate ≈ 3.0–5.9 METs, vigorous ≥ 6.0 METs) and used the midpoint of each range (e.g., 4.5 METs for moderate, 8.0 METs for vigorous) (24). For studies lacking specific intensity details, we assigned generic codes from the 2024 Physical Activity Compendium, selecting conservative estimates to minimize potential bias (e.g., general intensity for walking was 3.5 METs). For AE-RT, weekly doses of AE and RT were calculated separately and then summed.

**Table 2:** Definitions of different exercise modalities.

Exercise type	Definition
AE	AE refers to continuous or rhythmic physical activities that primarily rely on oxidative metabolism to sustain energy production and involve large muscle groups, leading to improvements in cardiorespiratory fitness. Typical examples include running, cycling, and walking.
RT	RT involves exercises designed to improve muscular strength, endurance, or power by working against external resistance, such as free weights, resistance bands, or body weight.
AE-RT	AE-RT refers to exercise programs that integrate both aerobic and resistance components within the same intervention period or training session, aiming to concurrently improve cardiorespiratory fitness and muscular strength.
Yoga	Yoga is a mind–body exercise practice that typically combines physical postures (asanas), breathing techniques (pranayama), and meditation or relaxation components, aiming to improve flexibility, balance, muscular strength, and psychological well being.

AE, aerobic exercise; RT, resistance training; AE-RT, combined aerobic and resistance training.

For studies reporting exercise attendance, the dose categorization was adjusted in cases of lower attendance to reflect the reduced actual exercise dose, since the amount of exercise delivered in interventions is often lower than the prescribed or intended dose (26). Based on the estimated METs-min/week, each intervention was assigned to the nearest predefined dose category. The interventions were grouped into six categores: 0 (usual care), 250, 500, 750, 1,000, and 1,250 METs-min/week. This categorization ensured adequate network connectivity, which is essential for conducting the network meta-analysis. For example, intensity of general yoga is 2.3 METs, and if the study reports that the exercise was performed five times a week for 60 min per session, the total exercise intensity would be calculated as follows: 2.3 (intensity) × 60 (min) × 5 (sessions) = 690 METs-min/week. If the study reports an average exercise session attendance rate of 70%, the dose would be adjusted to 690 × 0.7 = 483 METs-min/week. This adjusted dose would then be rounded to the nearest predefined dose category, which in this case would be 500 METs-min/week. The specific allocation process can be found in Supplementary Content 3.

### 2.5 Statistical analysis

#### 2.5.1 Pairwise meta-analyses

Given the use of various outcome measurement scales, standardized mean differences (SMD) were calculated based on the change from baseline to endpoint for both groups. For scales in which higher scores indicate worse outcomes, the MD were multiplied by −1 so that all outcomes were aligned in a consistent direction, with positive SMD reflecting improvement relative to the control group. Pairwise meta-analyses were conducted using Stata 16 to compare exercise with usual care for CRF in breast cancer survivors. A random-effects model was used when *I*^2^ > 25%. Statistical significance was determined when the 95% confidence interval (CI) did not include zero.

#### 2.5.2 Dose-response network meta-analysis

The dose-response analysis, exploring the relationship between exercise dose and different exercise types, was performed using the MBNMAdose package in *R* (*R* version 4.4.3, www.r-project.org). Dose-response curves were generated with the ggplot2 package. Following the default settings in MBNMAdose, we applied vague (non-informative) prior distributions to minimize bias and allow the data to guide the results. First, we assessed whether the analysis met the key assumptions of network meta-analysis, including network connectivity, data consistency, and network transitivity (27–29). Second, we tested several models to represent the dose-response relationships, including the emax, restricted cubic spline, log-linear, non-parametric, exponential, quadratic function, spline, and fractional polynomial models (30). The goodness of fit for the model was assessed using several criteria, including the deviance information criterion (DIC), standard deviation of residuals, model complexity, and residual deviation. For model selection, we followed previously published studies, which consistently chose the model with the lowest DIC (31–35). Given the anticipated heterogeneity, a random-effects model was used to account for potential variability across studies. The beta coefficient from the model was used to determine the effective and optimal exercise dose required for a significant reduction in CRF and to predict the ranking of different exercise modalities and doses based on their likelihood of inducing a change (20). Based on Bayesian dose-response analyses, significance is indicated when the 95% credible interval (CrI) of the effect estimate excludes zero.

#### 2.5.3 Additional analyses

Network meta-regressions were performed to examine whether various covariates significantly modified the intervention effects. The covariates of interest included average age (years), intervention length (weeks), and baseline fatigue status. Because the measurement scales and directionality of baseline fatigue differed across studies, baseline fatigue status was converted into a binary variable (severe fatigue vs. non-severe fatigue). The classification of severity was based on the definitions provided by each fatigue scale. For studies in which no explicit severity cut-offs were available, the total score was dichotomized at the median to define severe and non-severe fatigue. This harmonization was pragmatic rather than clinically standardized. Regression coefficients with 95% CrI were estimated to evaluate the influence of each covariate on CRF, with a CrI that crosses zero indicating no significant effect. Network meta-regressions were conducted using the R package MBNMAdose.

Subgroup analyses were performed to explore whether intervention effects differed according to baseline fatigue status (severe vs. non-severe) and intervention length (< 12 vs. ≥12 weeks), as these factors may contribute substantially to heterogeneity. The 12-week cut-off was chosen because it is commonly used to distinguish short- from long-term interventions and reflects a typical timeframe for physiological adaptation to exercise in intervention studies (36, 37). In addition, this threshold was selected because 12 weeks approximated the median intervention length among the included studies, helping to ensure a more balanced distribution of data and improved comparability between subgroups.

A sensitivity analysis was conducted by excluding studies with a high risk of bias to assess result variation. In addition, alternative pre–post correlation coefficients (*r* = 0.3 and 0.7) were applied to examine the impact of this assumption on the findings. Funnel plots, along with Begg's and Egger's tests, were used to evaluate publication bias and small-study effects (38, 39). Symmetry in the funnel plots and *p*-values >0.05 indicated no significant publication bias.

### 2.6 Risk of bias and certainty of evidence

Risk of bias was assessed using the Cochrane Risk of Bias tool (RoB 2), covering five domains: randomization, deviations from interventions, missing outcome data, outcome measurement, and selective reporting. Studies were classified as low risk, some concerns, or high risk (40). Two authors (DZL, ZYF) independently performed the assessment, with disagreements resolved by a third author (GP).

To evaluate the confidence in results, we used the CINeMA web application, which considers factors such as within-study bias, reporting bias, indirectness, imprecision, heterogeneity, and inconsistency (41). Evidence confidence was graded as high, moderate, low, or very low.

## 3 Results

### 3.1 Study selection

**Figure 1:**
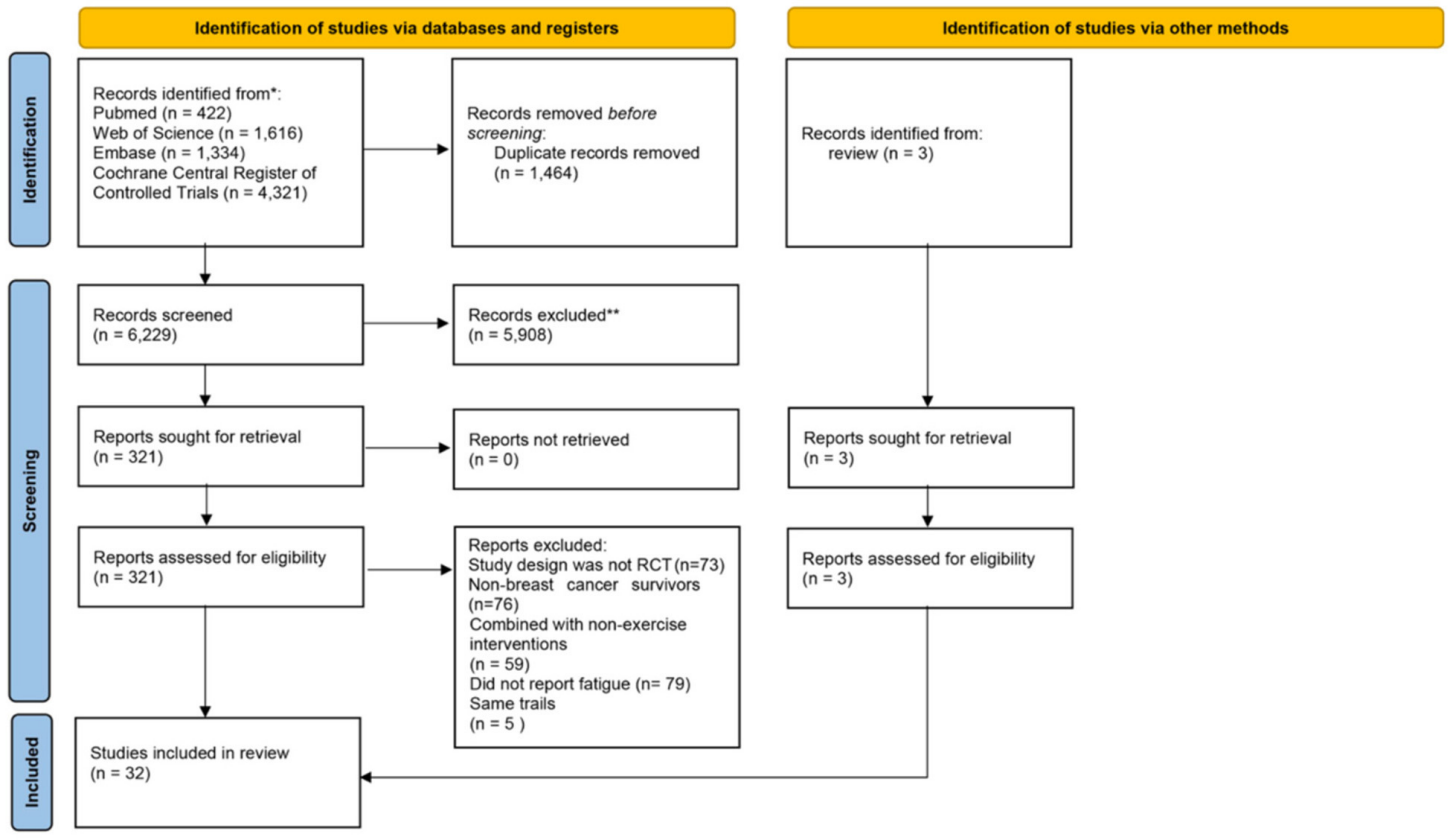
Screening and selection process of studies for the systematic review.

A total of 7,693 records were retrieved from four databases. After removing 1,464 duplicates, 6,229 records remained for title and abstract screening, of which 5,908 were excluded as irrelevant. The remaining 321 articles underwent full-text screening, leading to the exclusion of 292 studies based on the eligibility criteria. As a result, 29 studies were included in the systematic review. Additionally, 3 studies were identified through related review articles, bringing the total to 32 studies included in the final analysis. Figure 1 illustrates the screening and selection process.

### 3.2 Study characteristics

A total of 2,067 breast cancer survivors participated in the study, with 1,116 individuals (54%) in the exercise group and 951 in the usual care group (46%). The participants' mean ages ranged from 42.7 to 64.4 years, with an overall mean of 53.6 years. Participants had heterogeneous and mixed treatment histories (e.g., radiotherapy, chemotherapy, and surgery) prior to the exercise intervention. The interventions encompassed four exercise modalities: AE (10 arms), RT (7 arms), AE-RT (13 arms) and yoga (7 arms). All included studies were two-arm trials, except for one study with a three-arm design (42). Among the included studies, four used other exercise modalities as the control group (42–45), and two used different doses of the same exercise modality (46, 47), while the remaining studies employed usual care as the control condition. The exercise doses in the included studies ranged from 250 to 1,250 METs-min/week, with 500 METs-min/week representing the most common dose level (43.8%). The length of the interventions varied from 1 to 48 weeks, and the participants' cancer stages ranged from Stage 0 to Stage IV. Geographically, participants were recruited from Asia, Europe, the Americas, and Oceania, with the United States accounting for the largest proportion (33.3%). The Piper Fatigue Scale (PFS) was the most used assessment tool, utilized in 25% of the studies. Attendance rates for the exercise interventions ranged from 60.4 to 100%. No major exercise-induced adverse events were reported. Only five studies reported a total of 12 participants experiencing mild exercise-related pain. Detailed study characteristics are provided in Supplementary Content 4.

### 3.3 Risk of bias

Overall, Three studies were rated as high risk of bias, including those with no blinding of allocation personnel, a high proportion of missing outcome data, or missing registration numbers that precluded assessment of selective reporting bias. The remaining studies were rated as having “some concerns” due to unclear randomization methods, the inability to implement participant blinding, insufficient details on the handling of missing data, and the subjective nature of outcome measurements. Detailed risk-of-bias assessment criteria are provided in Supplementary Content 5.

### 3.4 Pairwise meta-analyses

**Figure 2:**
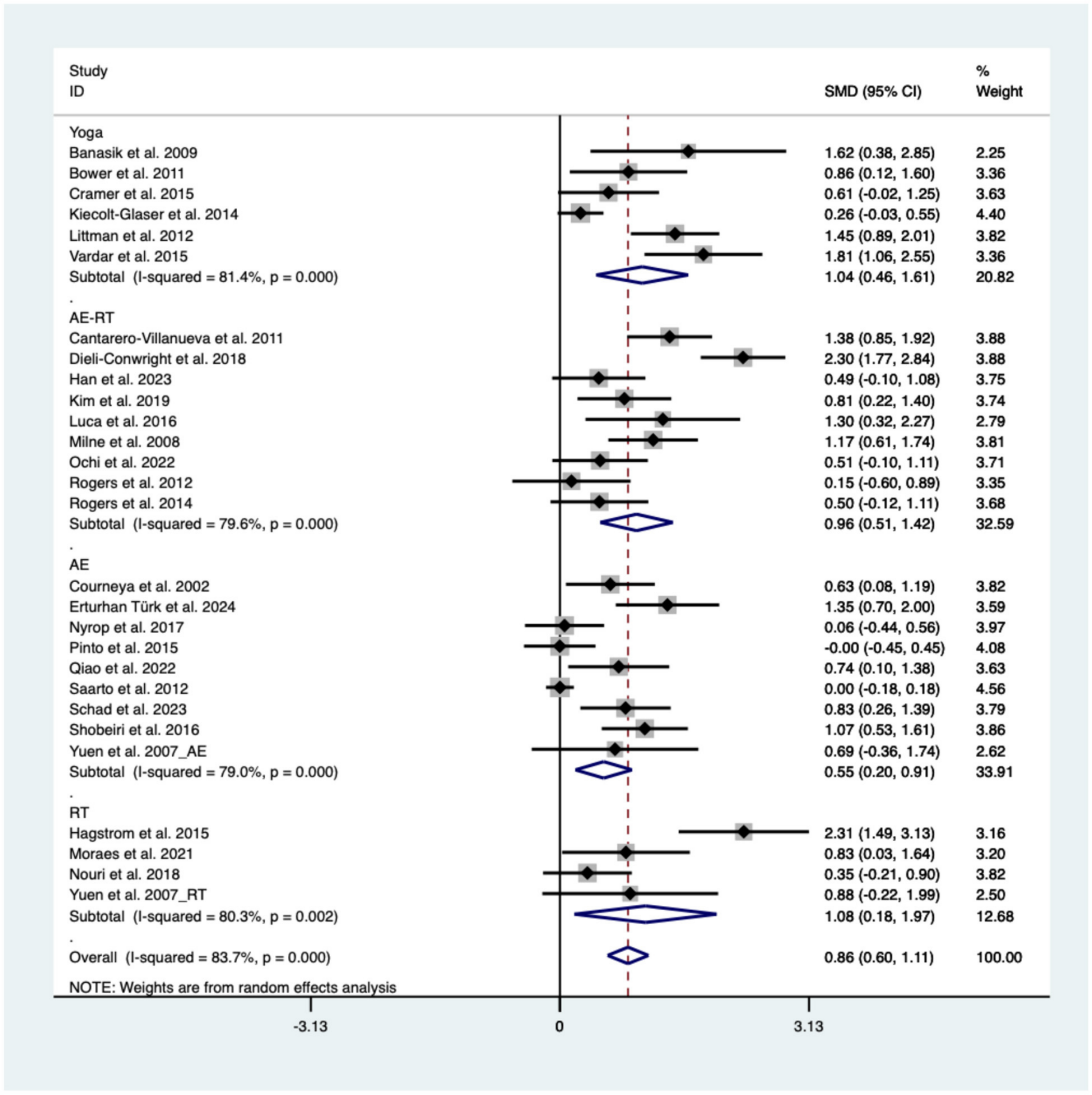
Forest plot of the effects of different exercise modalities on fatigue in breast cancer survivors. AE, aerobic exercise; AE-RT, combined aerobic and resistance training; RT, resistance training.

A total of 28 studies were included in the pairwise meta-analysis. Compared with usual care, exercise significantly improved CRF in breast cancer survivors (SMD = 0.86, 95% CI: 0.6, 1.11, *I*^2^ = 83.7%). By exercise modality, RT showed the greatest effect (SMD = 1.08, 95% CI: 0.18, 1.97, *I*^2^ = 80.3%) followed by yoga (SMD = 1.04, 95% CI: 0.46, 1.61, *I*^2^ = 81.4%), AE–RT (SMD = 0.96, 95% CI: 0.51, 1.42, *I*^2^ = 79.6%), and AE (SMD = 0.55, 95% CI: 0.2, 0.91, *I*^2^ = 79%). Figure 2 presents the pooled forest plot of the effects of different exercise modalities on CRF.

### 3.5 Dose–response meta-analysis

#### 3.5.1 Hypothesis testing and model selection

**Figure 3:**
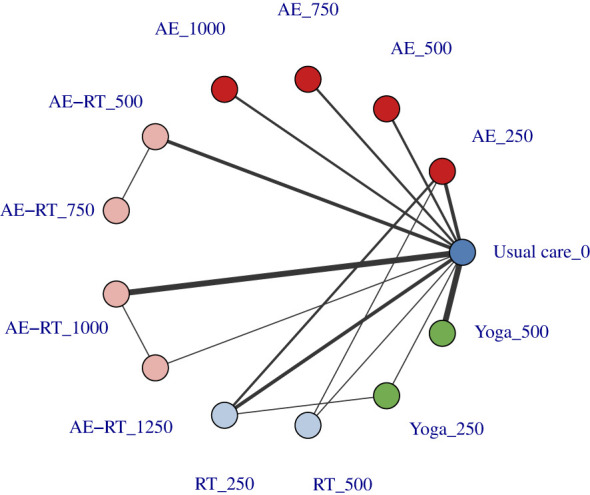
Connectivity of the dose-level network. The first value represents the specific intervention, and the second value corresponds to the dose (METs-min/week) of that intervention. Line thickness represents the number of studies available for comparison. AE, aerobic exercise; AE-RT, combined aerobic and resistance training; RT, resistance training.

No evidence was found to suggest that the dose-response network meta-analysis violated the assumptions of connectivity (Figure 3; Supplementary Content 6.1), consistency (Supplementary Content 6.2), or transitivity (Supplementary Content 6.3). Ultimately, the quadratic function which had the lowest DIC, was selected as the best-fitting approach (Supplementary Content 6.4).

#### 3.5.2 Dose-response relationship of overall exercise

**Figure 4:**
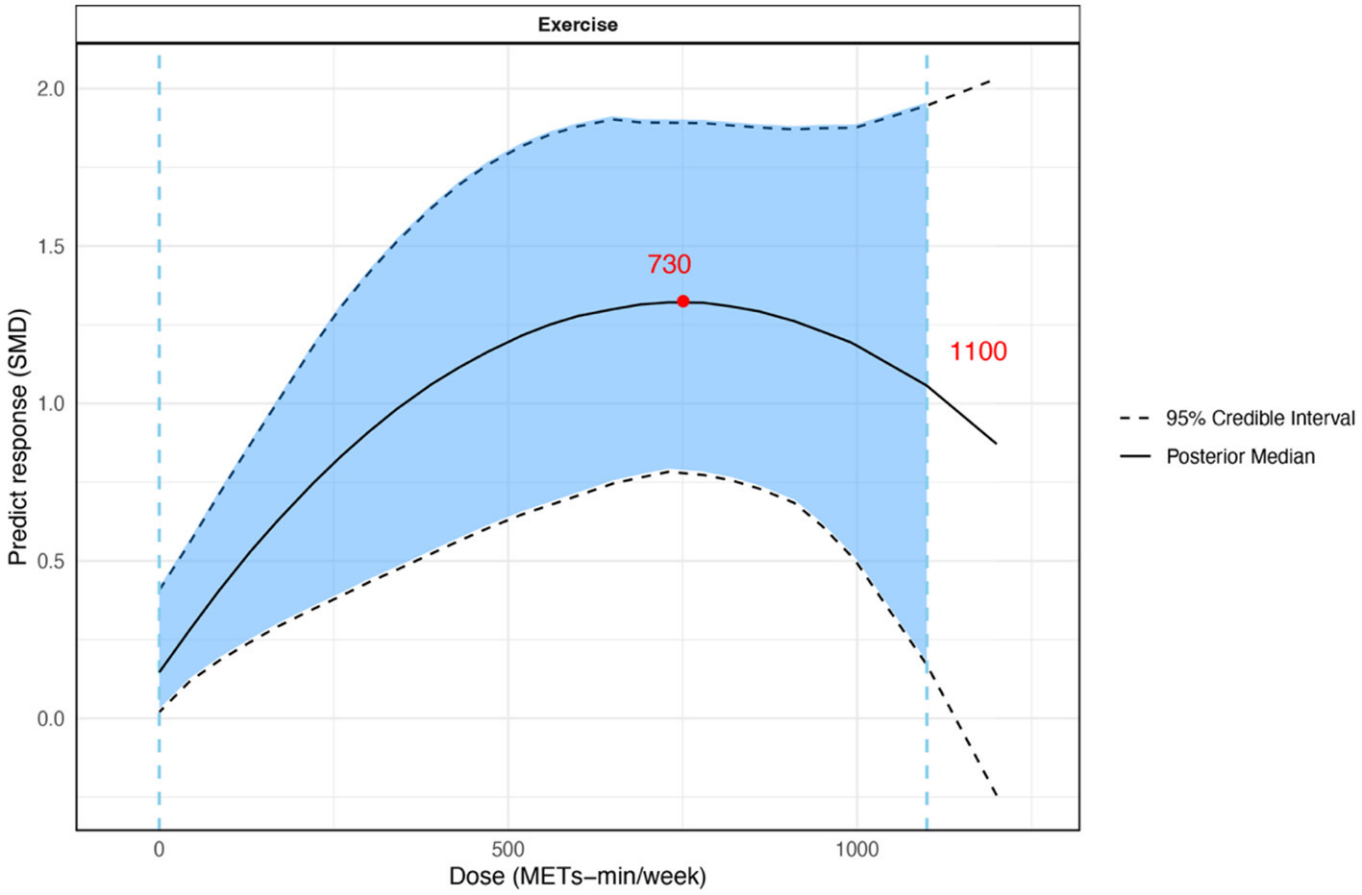
Dose–response relationship between total exercise (METs-min/week) and fatigue in breast cancer survivors. The blue dashed lines represent the minimum and maximum effective doses (1100 METs-min/week), the light blue shaded area indicates the significant region, and the red dots represent the optimal dose points (730 METs-min/week). METs-min/week, metabolic equivalent of task minutes per week

Due to methodological constraints, dose–response models cannot accommodate multiple arms within the same study with identical intervention–dose combinations. Therefore, three studies were excluded from the overall exercise analysis because they compared different exercise modalities at the same dose levels (42, 43, 45). Table 3 systematically presents the results of the dose–response network meta-analysis. Our analysis revealed an inverted U-shaped dose-response relationship between overall exercise dose and fatigue levels in breast cancer survivors. A minimum effective threshold could not be definitively established. As the exercise dose increased, the effects improved, peaking at 730 METs-min/week (SMD = 1.32, 95% CrI: 0.78, 1.89), after which the effects gradually declined. Beyond 1,100 METs-min/week, exercise ceased to be effective (Figure 4; Supplementary Content 7.1).

**Table 3:** Summary of dose-response network meta-analysis result.

Category	Data point	Effective dose (METs-min/week)	Optimal dose (METs-min/week)	SMD and 95% CrI at optimal dose
Overall exercise				
Exercise	58	~1,100	730	1.32 (0.78, 1.89)
Length (≥ 12 weeks)	38	~950	690	1.31 (0.46, 2.17)
Length (< 12 weeks)	20	430–1,200	1,200	1.38 (0.23, 2.58)
Fatigue status (non-severe)	26	~1,000	690	0.63 (0.29, 1.04)
Fatigue status (severe)	32	~1,100	820	1.87 (0.82, 2.9)
Sensitivity analysis				
Excluding high risk bias studies	52	~1,100	820	1.27 (0.69, 1.85)
*R* = 0.7	58	~1,100	730	1.32 (0.73, 1.94)
*R* = 0.3	58	~1,100	730	1.32 (0.82, 1.97)
Exercise type				
AE	19	~830	590	0.75 (0.15, 1.37)
AE-RT	22	~1,200	1,200	1.44 (0.63, 2.21)
RT	11	~450	290	1.25 (0.54, 1.97)
Yoga	13	~500	340	1.28 (0.33, 2.24)

AE, aerobic exercise; AE-RT, combined aerobic and resistance training; RT, resistance training; METs-min/week, metabolic equivalents of task minutes per week; SMD, standard mean difference.

Subgroup analysis showed that for interventions length ≥12 weeks, the effective dose ranged from 0 to 950 METs-min/week, with the greatest effect at 690 METs-min/week (SMD =1.31, 95% CrI: 0.46, 2.17). For interventions length < 12 weeks, the effective dose ranged from 430 to 1,200 METs-min/week, with the greatest effect at 1,200 METs-min/week (SMD = 1.38, 95% CrI: 0.23, 2.58). In addition, exercise showed greater effects in individuals with severe fatigue, with the greatest effect observed at 820 METs-min/week (SMD = 1.87, 95% CrI: 0.82, 2.9). In contrast, among individuals with non-severe fatigue, the greatest effect was observed at 660 METs-min/week (SMD = 0.63, 95% CrI: 0.29, 1.04).

After excluding studies with a high risk of bias in the sensitivity analysis, the effective exercise dose range (0–1,100 MET-min/week) and the optimal dose (730 MET-min/week) remained unchanged, although the effect size was slightly attenuated (SMD = 1.27, 95% CrI: 0.69, 1.85). Sensitivity analyses using correlation coefficients of 0.3 and 0.7 yielded consistent results, with exercise remaining significantly effective (Table 3).

#### 3.5.3 Dose-response relationship of different exercise modalities

**Figure 5:**
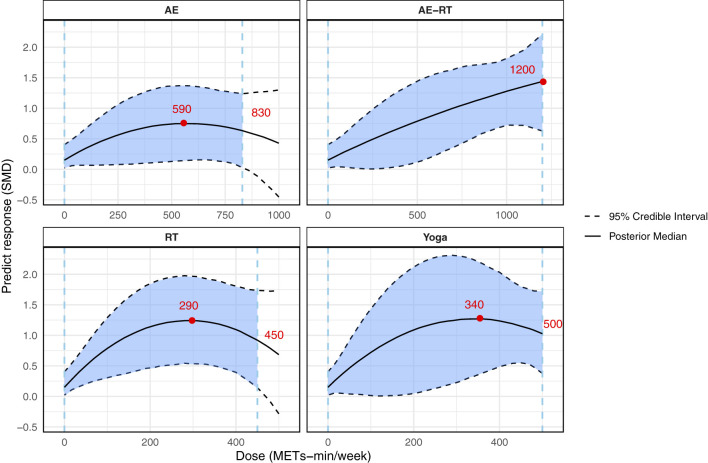
Dose–response relationship between different exercise modalities (METs-min/week) and fatigue in breast cancer survivors. The blue dashed lines represent the minimum and maximum effective doses, the blue shaded area indicates the significant region, and the red dots represent the optimal dose points. METs-min/week, metabolic equivalent of task minutes per week; AE, aerobic exercise; AE-RT, combined aerobic and resistance training; RT, resistance training.

Figure 5 shows the dose-response relationship for the four exercise modalities. Significant dose–response relationships were observed between AE, RT, AE–RT, yoga, and CRF. Specifically, for AE, the maximum effective dose was 830 METs-min/week, with the optimal dose at 590 METs-min/week (SMD = 0.75, 95% CrI: 0.15, 1.37). For AE–RT, both the maximum effective dose and the optimal dose were observed at 1,200 METs-min/week (SMD = 1.44, 95% CrI: 0.63, 2.21). For RT, the maximum effective dose was 450 METs-min/week, with the optimal dose at 290 METs-min/week (SMD = 1.25, 95% CrI: 0.54, 1.97). For yoga, the maximum effective dose was 500 METs-min/week, with the greatest effect observed at 340 METs-min/week (SMD = 1.28, 95% CrI: 0.33, 2.24). The overall predicted ranking of exercise modalities and doses indicated that AE-RT at 1200 METs-min/week was the most effective (Supplementary Content 7.2).

#### 3.5.4 Meta-regression analyses

Table 4 presents the results of the meta-regression analyses. No significant moderating effects of age or intervention length on CRF were identified. However, compared with non-severe fatigue, severe baseline fatigue was associated with significantly greater improvements in CRF (SMD = 1.51, 95% CrI: 0.09, 2.51).

**Table 4:** Dose-response network meta-regression.

Covariate	DIC	Residual deviance	Shared beta (median and 95% CrI)	SD
Age (years)	344.5	215.2	−0.25 (−0.53, 0.04)	0.45 (0.28, 0.78)
Intervention length (weeks)	737.4	594.3	−0.01 (−0.23, 0.25)	0.34 (0.16, 0.72)
Baseline fatigue status (non severe/severe)	368	238.2	1.51 (0.09, 2.51)	2.11 (1.35, 3.74)

DIC, deviance information criterion; SD, standard deviation.

### 3.6 Publication bias and certainty of evidence

Supplementary Content 8 presents a detailed analysis of publication bias. The funnel plot appeared symmetrical, and both Egger's and Begg's tests did not indicate significant publication bias (*p* > 0.05), suggesting that the results are unlikely to be substantially influenced by publication bias.

The CINeMA evidence rating revealed that within-study bias, heterogeneity, and incoherence across the four exercise interventions resulted in low or very low certainty of evidence: yoga, RT, AE-RT were rated as low certainty; while AE were rated as very low certainty (Supplementary Content 9).

## 4 Discussion

### 4.1 Summary and comparison

This study explores the dose-response relationship of different exercise modalities on fatigue in breast cancer survivors and to propose optimized exercise prescriptions based on the evidence. It holds two key clinical implications. First, clinicians may refer to our findings when developing personalized exercise prescriptions tailored to individual survivor characteristics for the management of CRF. Second, future exercise intervention studies can refer to our dose-range framework to develop CRF-specific programs and further validate the effectiveness of these dose ranges, thereby strengthening the evidence base in this field.

The results indicate an inverted U-shaped dose-response relationship between overall exercise dose and CRF in breast cancer survivors. This suggests that once the exercise dose reaches a certain threshold, it may not only fail to improve fatigue but may even exacerbate it, consistent with the physiological relationship between exercise and fatigue. When stratified by exercise modality, it should be noted that the relative effectiveness of exercise modalities differed between the pairwise meta-analysis and the dose–response network meta-analysis. This difference may be explained by the fact that pairwise meta-analysis does not incorporate studies direct comparisons between exercise modalities and cannot account for dose effects, whereas the dose-response network meta-analysis simultaneously integrates both factors. In this context, the results from the network meta-analysis may provide more informative evidence for comparative effectiveness. The comprehensive ranking indicates that AE-RT at 1,200 MET-min/week is the most effective modality and dose for alleviating fatigue in breast cancer survivors; however, this estimate reflects relative model-based probabilities rather than a definitive clinical hierarchy and should be interpreted cautiously given variability in the quantity and quality of the underlying evidence. Notably, both meta-regression and subgroup analyses consistently showed that baseline fatigue severity in breast cancer survivors may significantly contribute to heterogeneity in the evidence. Subgroup analyses indicated that interventions length less than 12 weeks were more effective, possibly due to physiological adaptations from long-term exercise and reduced adherence over time. However, these findings should not be considered definitive and warrant further investigation and validation. The mechanisms underlying exercise effects on CRF are complex. Cancer treatments can induce repeated elevations of pro-inflammatory cytokines, triggering compensatory increases in anti-inflammatory cytokines and resulting in maladaptive chronic peripheral inflammation (48, 49). This peripheral inflammation can disrupt the blood–brain barrier (50). Exercise may help restore blood–brain barrier integrity by normalizing permeability and reestablishing tight junction protein expression (51). Evidence from a multiple sclerosis mouse model shows that endurance exercise reduces central IFN-γ and IL-1β production, protecting the blood–brain barrier (51). By preserving blood–brain barrier function, exercise may limit the entry of neurotoxic metabolites into the central nervous system, potentially alleviating CRF (52).

Regarding exercise modality, differences in effectiveness were observed across interventions, with AE-RT, RT, and yoga showing relatively greater improvements, while AE alone demonstrated comparatively smaller effects. Previous network meta-analyses identifying yoga as the most effective modality for alleviating CRF in patients undergoing primary treatment (53). Additionally, a meta-analysis also highlighted yoga as the most effective exercise for alleviating CRF after cancer treatment (54). Future studies with larger sample sizes and more rigorously designed RCTs are needed to validate the effectiveness of yoga and further assess the optimal and effective doses we recommend. We attempted to explain the mechanisms behind yoga's effectiveness in alleviating CRF. As a low-intensity exercise, yoga combines physical postures with mindful breathing, making it particularly well-suited to the physical and mental needs of cancer patients. Research has shown that yoga can reduce stress by lowering cortisol levels and increasing dehydroepiandrosterone sulfate levels, thereby helping to regulate hypothalamic-pituitary-adrenal axis dysfunction (55). Additionally, yoga has been shown to reduce tumor necrosis factor-α (TNF-α) a biomarker that may be associated with fatigue (55). Future research is needed to further explore the relationship between yoga and the inflammatory markers associated with CRF.

AE, such as walking, running and cycling, which generally have a higher intensity than yoga, can enhance cardiovascular function and improve cells' ability to utilize oxygen. A previous overview of systematic reviews, which included 29 studies, assessed the effects of exercise on the breast cancer population (56). The review found that yoga and AE significantly improved fatigue in breast cancer patients, which is consistent with our findings (56). In addition, the study found that improved aerobic endurance was associated with better quality of life, reduced fatigue, and lower levels of depression and anxiety, suggesting it may directly influence patients' self-reported treatment outcomes (57). This factor is also a significant predictor of disease progression and mortality (58). Furthermore, high-intensity AE may alleviate CRF by reducing interleukin-6 levels and T-cell surface glycoproteins (59).

Previous studies suggest that AE-RT may be among the most effective interventions for reducing inflammation in cancer populations (60), and that both higher-intensity and longer-duration exercise (>280 min/week) may confer greater anti-inflammatory effects (61). Interestingly, our findings indicate that AE-RT at a higher weekly volume (approximately 1,200 MET-min/week) is associated with the greatest reduction in CRF. Taken together, these findings raise the possibility that improvements in inflammatory status and fatigue may be related. However, further research is required to disentangle the independent effects of exercise intensity and volume on these outcomes. Previous studies have shown that RT can significantly alleviate fatigue in cancer patients undergoing treatment (62), and our findings are consistent with these results. Cancer type and treatment status may not substantially modify the effects of RT on fatigue. RT may potentially modulate the neural mechanisms of inflammation by improving kynurenine metabolism, further alleviating fatigue (62, 63).

### 4.2 Clinical recommendations

Current guidelines recommend that cancer populations engage in 150 min moderate intensity of AE per week, supplemented by a few sessions of RT to alleviate CRF (5). However, this recommendation largely follows the World Health Organization's (WHO) general exercise guidelines for healthy adults (64). Unlike healthy adults, cancer survivors typically have reduced exercise capacity, presenting challenges in balancing feasibility with effectiveness. It should be noted that the model-identified optimal dose for AE-RT (1,200 METs-min/week) corresponds to the upper bound of the WHO-recommended range (600–1,200 METs-min/week) and may exceed the capacity of many cancer survivors. In comparison, yoga may represent a more feasible alternative, as it may achieve comparable improvements at lower exercise doses. However, it should be noted that long-term yoga may have negative effects for individuals with a history of back pain (65, 66). Although RT was associated with relatively large effects, it should be noted that excessive RT or insufficient recovery may increase physical pain, potentially exacerbating fatigue (67, 68). AE demonstrated smaller effects than other exercise modalities in both pairwise and dose–response meta-analyses, with very low certainty of evidence. Further large-scale studies are needed to better establish its effects on fatigue in breast cancer survivors. Overall, these recommendations are derived from the findings of the present study and should be interpreted as model-informed, rather than prescriptive. Exercise prescriptions should ultimately be individualized based on patients' tolerance and responses to different exercise modalities and doses.

### 4.3 Strengths and limitations

Our work offers several notable advantages. (1) It incorporates trials that directly compare different doses within the same exercise modality, whereas previous meta-analyses have primarily focused on comparisons across different interventions and often overlooked such within-modality evidence. (2) It explicitly models nonlinear dose–response relationships. In our study, exercise doses were divided into multiple average dose intervals, which can help overcome some limitations of subgroup analyses and meta-regression, as these approaches often rely on dichotomized or linear assumptions. (3) it quantifies exercise dose using MET-minutes per week, simultaneously accounting for intensity, duration, and frequency, whereas subgroup analyses typically control only a single variable—such as duration—implicitly assuming equal intensity and frequency, which may obscure true differences in total exercise exposure. (4) The methodological approach was comprehensive, using pairwise meta-analyses, multiple sensitivity analyses, subgroup analyses, and meta-regression to explore potential sources of heterogeneity and assess the robustness of the findings.

Although our study has several strengths, the following limitations need to be acknowledged. (1) This study focused exclusively on breast cancer survivors who had completed primary treatment, and therefore the findings cannot be generalized to individuals with other cancer types or those undergoing active treatment. (2) Continuous exercise doses (METs-min/week) were categorized into discrete levels to ensure sufficient data at each dose point and maintain network connectivity. This discretization may introduce exposure misclassification and reduce the precision of detecting nonlinear dose–response relationships, and thus the corresponding dose–response results should be interpreted with caution. (3) While using METs to assess exercise dose offers certain advantages, such as standardizing various exercise types into a fixed intensity scale and aiding in the calculation of total exercise dose, it also has significant limitations. First, METs calculations are based on the average resting metabolic rate, which overlooks individual factors such as age, gender, weight, and physical condition—each of which affects energy expenditure. Additionally, METs assumes a uniform resting metabolic rate (1 METs) for all individuals, ignoring personal variations. This method is also less suitable for exercises with fluctuating intensities and is challenging to measure accurately without specialized equipment. Furthermore, standard MET values do not account for health conditions that may alter an individual's response to exercise. (4) Although we explored potential sources of heterogeneity through subgroup analyses and meta-regression, differences in cancer stage and prior treatment modalities among the included studies could not be examined, as the original studies did not provide data stratified by these factors. Future studies are encouraged to report stage- and treatment-specific data to enable a more precise evaluation of their potential impact on intervention effects. In addition, dichotomization may result in a coarse representation of related variables, such as fatigue severity and intervention length. Therefore, related findings should be interpreted with caution. (5) The certainty of evidence for several exercise types was rated as low or very low. Although this does not directly affect our dose–response findings, it highlights that our results are based on a relatively limited evidence base.

## 5 Conclusions

This study provides exploratory dose–response evidence on the effects of exercise in alleviating CRF among breast cancer survivors after primary treatment. Exercise is associated with a reduction in CRF among breast cancer survivors in an inverted U-shaped manner; a minimum effective threshold could not be definitively established, with a peak effect at 730 METs-min/week, and effects were no longer significant beyond 1,100 METs-min/week. Regarding exercise modalities, AE (0–830 METs-min/week), AE-RT (0–1,200 METs-min/week), RT (0–450 METs-min/week) and yoga (0–500 METs-min/week) were significantly associated with CRF reduction. However, given the low and very low certainty of the evidence, it is still premature to directly apply these findings to clinical practice. Future high-quality randomized controlled trials are warranted to further confirm these results and validate the recommended dose ranges.

## Data Availability

The original contributions presented in the study are included in the article/Supplementary material, further inquiries can be directed to the corresponding author.
