# Supplementary material for: Optimized exercise prescription for alleviating cancer-related fatigue in breast cancer survivors after primary treatment: a systematic review and dose-response network meta-analysis
Source: Front Public Health. 2026 May 15;14:1795975. doi: 10.3389/fpubh.2026.1795975 (PMC13218988; doi:10.3389/fpubh.2026.1795975)
Supplement: Supplementary file 1 [file Data_Sheet_1.pdf]

Supplementary Content

1. PRISMA NMA CHECKLIST **2**

2. SEARCH STRATEGY **8**

2.1 PUBMED 8
2.2 WEB OF SCIENCE 8
2.3 EMBASE 9
2.4 COCHRANE 9

3. ENCODING EXAMPLE **11**

4. TABLE S2. STUDY CHARACTERISTICS **12**

5. RISK OF BIAS **15**

6. DOSE-RESPONSE NETWORK META-ANALYSES **17**

6.1 CONNECTIVITY 17
6.2 CONSISTENCY 17
6.3 TRANSITIVITY 18
6.4 MODELS' SELECTION 19
6.4.1 Linear and non-linear functions and models fit comparison *19*
6.4.2. Models fit comparison *21*
6.4.3 Table S5. Models fit comparison *24*

7. DOSE-RESPONSE RELATIONSHIPS **26**

7.1 DOSE-RESPONSE RELATIONSHIP BETWEEN EXERCISE DOSE AND FATIGUE 26
7.2 EFFECTIVENESS RANKING OF DIFFERENT EXERCISES AND DOSES 27

8. PUBLICATION BIAS **30**

9. GRADE SUMMARY OF ALL STUDIES **31**

9.1 WITHIN-STUDY BIAS 31
9.2 REPORTING BIAS (PUBLICATION BIAS AND SELECTIVE REPORTING) 31
9.3 INDIRECTNESS 31
9.4 IMPRECISION 31
9.5 HETEROGENEITY 31
9.6 INCOHERENCE 31
9.7 SUMMARIZING JUDGMENTS ACROSS CINEMA DOMAINS 31

REFERENCES **33**

1. PRISMA NMA Checklist

PRISMA NMA Checklist of Items to Include When Reporting A Systematic Review Involving a Network Meta-analysis

Section/Topic	Item #	Checklist Item	Reported on Section
**TITLE**			
Title	1	Identify the report as a systematic review *incorporating a network meta-analysis (or related form of meta-analysis).*	**Title**
**ABSTRACT**			
Structured summary	2	Provide a structured summary including, as applicable:<br>**Background:** main objectives<br>**Methods:** data sources; study eligibility criteria, participants, and interventions; study appraisal; and *synthesis methods, such as network meta-analysis.*<br>**Results:** number of studies and participants identified; summary estimates with corresponding confidence/credible intervals; *treatment rankings may also be discussed. Authors may choose to summarize pairwise comparisons against a chosen treatment included in their analyses for brevity.*<br>**Discussion/Conclusions:** limitations; conclusions and implications of findings.<br>**Other:** primary source of funding; systematic review registration number with registry name.	**Abstract**
**INTRODUCTION**			
Rationale	3	Describe the rationale for the review in the context of what is already known*, including mention of why a network meta-analysis has been conducted.*_	**Introduction**
Objectives	4	Provide an explicit statement of questions being addressed, with reference to participants, interventions, comparisons, outcomes, and study design (PICOS).	**Introduction**

METHODS

Protocol and registration	5	Indicate whether a review protocol exists and if and where it can be accessed (e.g., Web address); and, if available, provide registration information, including registration number.	**Protocol and registration**
Eligibility criteria	6	Specify study characteristics (e.g., PICOS, length of follow-up) and report characteristics (e.g., years considered, language, publication status) used as criteria for eligibility, giving rationale. *Clearly describe eligible treatments included in the treatment network, and note whether any have been clustered or merged into the same node (with justification).*_	Eligibility criteria
Information sources	7	Describe all information sources (e.g., databases with dates of coverage, contact with study authors to identify additional studies) in the search and date last searched.	**Search strategy**
Search	8	Present full electronic search strategy for at least one database, including any limits used, such that it could be repeated.	**Search strategy;** Supplementray Content 2
Study selection	9	State the process for selecting studies (i.e., screening, eligibility, included in systematic review, and, if applicable, included in the meta-analysis).	**Search strategy**
Data collection process	10	Describe method of data extraction from reports (e.g., piloted forms, independently, in duplicate) and any processes for obtaining and confirming data from investigators.	**Data extraction and coding**
Data items	11	List and define all variables for which data were sought (e.g., PICOS, funding sources) and any assumptions and simplifications made.	**Data extraction and coding**
**Geometry of the network**	**S1**	Describe methods used to explore the geometry of the treatment network under study and potential biases related to it. This should include how the evidence base has been graphically summarized for presentation, and what characteristics were compiled and used to describe the evidence	**Dose-response network meta-analysis**

		base to readers.	
Risk of bias within individual studies	12	Describe methods used for assessing risk of bias of individual studies (including specification of whether this was done at the study or outcome level), and how this information is to be used in any data synthesis.	**Risk of bias and certainty of evidence**
Summary measures	13	State the principal summary measures (e.g., risk ratio, difference in means). *Also describe the use of additional summary measures assessed, such as treatment rankings and surface under the cumulative ranking curve (SUCRA) values, as well as modified approaches used to present summary findings from meta-analyses.*	**Dose-response network meta-analysis**
Planned methods of analysis	14	Describe the methods of handling data and combining results of studies for each network meta-analysis. This should include, but not be limited to:<br>• *Handling of multi-arm trials;*<br>• *Selection of variance structure;*<br>• *Selection of prior distributions in Bayesian analyses; and*<br>• *Assessment of model fit.*	**Statistical analysis**
**Assessment of Inconsistency**	**S2**	Describe the statistical methods used to evaluate the agreement of direct and indirect evidence in the treatment network(s) studied. Describe efforts taken to address its presence when found.	**Dose-response network meta-analysis**
Risk of bias across studies	15	Specify any assessment of risk of bias that may affect the cumulative evidence (e.g., publication bias, selective reporting within studies).	**Risk of bias and certainty of evidence**
Additional analyses	16	Describe methods of additional analyses if done, indicating which were pre-specified. This may include, but not be limited to, the following:<br>• Sensitivity or subgroup analyses;<br>• Meta-regression analyses;<br>• *Alternative formulations of the treatment network; and*<br>• *Use of alternative prior distributions*	**Additional analyses**

		for Bayesian analyses (if applicable).	

RESULTS†

Study selection	17	Give numbers of studies screened, assessed for eligibility, and included in the review, with reasons for exclusions at each stage, ideally with a flow diagram.	**Study selection**
**Presentation of network structure**	**S3**	Provide a network graph of the included studies to enable visualization of the geometry of the treatment network.	**Figure 3**
**Summary of network geometry**	**S4**	Provide a brief overview of characteristics of the treatment network. This may include commentary on the abundance of trials and randomized patients for the different interventions and pairwise comparisons in the network, gaps of evidence in the treatment network, and potential biases reflected by the network structure.	**Supplementary content 6.1**
Study characteristics	18	For each study, present characteristics for which data were extracted (e.g., study size, PICOS, follow-up period) and provide the citations.	**Study characteristics**
Risk of bias within studies	19	Present data on risk of bias of each study and, if available, any outcome level assessment.	**Risk of bias**
Results of individual studies	20	For all outcomes considered (benefits or harms), present, for each study: 1) simple summary data for each intervention group, and 2) effect estimates and confidence intervals. *Modified approaches may be needed to deal with information from larger networks.*	**Pairwise meta-analyses; Dose – response meta-analysis**
Synthesis of results	21	Present results of each meta-analysis done, including confidence/credible intervals. *In larger networks, authors may focus on comparisons versus a particular comparator (e.g. placebo or standard care), with full findings presented in an appendix. League tables and forest plots may be considered to summarize pairwise comparisons.* If additional summary measures were explored	**Pairwise meta-analyses; Dose – response meta-analysis;**

		(such as treatment rankings), these should also be presented.	
**Exploration for inconsistency**	**S5**	Describe results from investigations of inconsistency. This may include such information as measures of model fit to compare consistency and inconsistency models, *P* values from statistical tests, or summary of inconsistency estimates from different parts of the treatment network.	**Supplementray Contents 6.2-6.3**
Risk of bias across studies	22	Present results of any assessment of risk of bias across studies for the evidence base being studied.	**Publication bias and certainty of evidence**
Results of additional analyses	23	Give results of additional analyses, if done (e.g., sensitivity or subgroup analyses, meta-regression analyses*, alternative network geometries studied, alternative choice of prior distributions for Bayesian analyses,* and so forth).	**Meta-regression analyses**
**DISCUSSION**			
Summary of evidence	24	Summarize the main findings, including the strength of evidence for each main outcome; consider their relevance to key groups (e.g., healthcare providers, users, and policy-makers).	**Summary and comparison**
Limitations	25	Discuss limitations at study and outcome level (e.g., risk of bias), and at review level (e.g., incomplete retrieval of identified research, reporting bias). *Comment on the validity of the assumptions, such as transitivity and consistency. Comment on any concerns regarding network geometry (e.g., avoidance of certain comparisons).*	**Strengths and limitations**
Conclusions	26	Provide a general interpretation of the results in the context of other evidence, and implications for future research.	**Conclusions**
**FUNDING**			
Funding	27	Describe sources of funding for the systematic review and other support (e.g., supply of data); role of funders for the systematic review. This should also include	**NA**

		information regarding whether funding has been received from manufacturers of treatments in the network and/or whether some of the authors are content experts with professional conflicts of interest that could affect use of treatments in the network.	

PICOS = population, intervention, comparators, outcomes, study design.
* Text in italics indicateS wording specific to reporting of network meta-analyses that has been added to guidance from the PRISMA statement.
† Authors may wish to plan for use of appendices to present all relevant information in full detail for items in this section.

2. Search strategy

2.1 Pubmed

("Exercise"[Mesh] OR "Exercise Therapy"[Mesh] OR "Physical Fitness"[Mesh] OR "Motor Activity"[Mesh] OR "Sports"[Mesh] OR "Resistance Training"[Mesh] OR "Yoga"[Mesh] OR "Tai Ji"[Mesh] OR "Dancing"[Mesh] OR "Walking"[Mesh] OR "Physical Exertion"[Mesh] OR "physical activity"[Title/Abstract] OR "movement"[Title/Abstract] OR "fitness"[Title/Abstract] OR "aerobic exercise"[Title/Abstract] OR "resistance training"[Title/Abstract] OR "strength training"[Title/Abstract] OR "endurance exercise"[Title/Abstract] OR "yoga"[Title/Abstract] OR "tai chi"[Title/Abstract] OR "pilates"[Title/Abstract] OR "dance"[Title/Abstract] OR "walking"[Title/Abstract] OR "jogging"[Title/Abstract] OR "stretching"[Title/Abstract] OR "mind-body exercise"[Title/Abstract])

AND

("Breast Neoplasms"[Mesh] OR "Breast Neoplasm"[Title/Abstract] OR "Neoplasm, Breast"[Title/Abstract] OR "Neoplasms, Breast"[Title/Abstract] OR "Breast Tumors"[Title/Abstract] OR "Breast Tumor"[Title/Abstract] OR "Tumor, Breast"[Title/Abstract] OR "Tumors, Breast"[Title/Abstract] OR "Breast Cancer"[Title/Abstract])

AND

("Fatigue"[Mesh] OR "fatigue"[Title/Abstract] OR "fatigue syndrome"[Title/Abstract] OR "tiredness"[Title/Abstract] OR "exhaustion"[Title/Abstract] OR "energy depletion"[Title/Abstract] OR "physical fatigue"[Title/Abstract])

AND

("Randomized Controlled Trial"[Title/Abstract] OR "RCT"[Title/Abstract] OR "randomized trial"[Title/Abstract] OR "controlled clinical trial"[Title/Abstract] OR "randomized study"[Title/Abstract] OR "randomized controlled trial"[Title/Abstract] OR "randomized clinical trial"[Title/Abstract] OR "RCTs"[Title/Abstract] OR "randomized clinical studies"[Title/Abstract] OR "intervention trial"[Title/Abstract] OR "randomized intervention"[Title/Abstract] OR "trial"[Title/Abstract] OR "experimental study"[Title/Abstract])

2.2 Web of Science

TS=(exercise OR physical activity OR movement OR fitness OR aerobic exercise OR resistance training OR strength training OR endurance exercise OR yoga OR tai chi OR pilates OR dance OR walking OR jogging OR stretching OR mind-body exercise OR sports OR physical exertion OR dancing OR "tai ji" OR "exercise therapy" OR "motor activity" OR "physical fitness")

AND

TS=(breast cancer OR breast neoplasm OR neoplasm, breast OR neoplasms, breast OR breast tumors OR breast tumor OR tumor, breast OR tumors, breast OR breast neoplasms)

AND

TS=(fatigue OR fatigue syndrome OR tiredness OR exhaustion OR energy depletion OR physical fatigue)

AND
TS=(randomized controlled trial OR RCT OR randomized trial OR controlled clinical trial OR randomized study OR randomized clinical trial OR RCTs OR randomized clinical studies OR intervention trial OR randomized intervention OR trial OR experimental study)

2.3 Embase

('exercise'/exp OR 'exercise therapy':ti,ab OR 'physical fitness':ti,ab OR
'motor activity':ti,ab OR 'sports':ti,ab OR 'resistance training':ti,ab OR
'yoga':ti,ab OR 'tai ji':ti,ab OR 'dancing':ti,ab OR 'walking':ti,ab OR
'physical exertion':ti,ab OR 'physical activity':ti,ab OR 'movement':ti,ab OR
'fitness':ti,ab OR 'aerobic exercise':ti,ab OR 'strength training':ti,ab OR
'endurance exercise':ti,ab OR 'tai chi':ti,ab OR 'pilates':ti,ab OR 'dance':ti,ab OR
'jogging':ti,ab OR 'stretching':ti,ab OR 'mind-body exercise':ti,ab)
AND
('breast neoplasms'/exp OR 'breast neoplasm':ti,ab OR 'neoplasm, breast':ti,ab OR
'neoplasms, breast':ti,ab OR 'breast tumors':ti,ab OR 'breast tumor':ti,ab OR
'tumor, breast':ti,ab OR 'tumors, breast':ti,ab OR 'breast cancer':ti,ab)
AND
('fatigue'/exp OR 'fatigue':ti,ab OR 'fatigue syndrome':ti,ab OR 'tiredness':ti,ab OR
'exhaustion':ti,ab OR 'energy depletion':ti,ab OR 'physical fatigue':ti,ab)
AND
('randomized controlled trial'/exp OR 'randomized controlled trial':ti,ab OR
'rct':ti,ab OR 'randomized trial':ti,ab OR 'controlled clinical trial':ti,ab OR
'randomized study':ti,ab OR 'randomized clinical trial':ti,ab OR 'rcts':ti,ab OR
'randomized clinical studies':ti,ab OR 'intervention trial':ti,ab OR 'randomized intervention':ti,ab OR
'trial':ti,ab OR 'experimental study':ti,ab)

2.4 Cochrane

#1 MeSH descriptor: [Exercise] explode all trees
#2 physical activity
#3 exercise
#4 movement
#5 fitness
#6 aerobic exercise
#7 resistance training
#8 strength training
#9 endurance exercise
#10 yoga
#11 tai chi
#12 pilates
#13 dance
#14 walking
#15 jogging

#16 stretching
#17 mind-body exercise
#18 sports
#19 physical exertion
#20 #1 OR #2 OR #3 OR #4 OR #5 OR #6 OR #7 OR #8 OR #9 OR #10 OR #11 OR #12 OR #13 OR #14 OR #15 OR #16 OR #17 OR #18 OR #19
#21 MeSH descriptor: [Breast Neoplasms] explode all trees
#22 Breast Neoplasm
#23 Neoplasm, Breast
#24 Neoplasms, Breast
#25 Breast Tumors
#26 Breast Tumor
#27 Tumor, Breast
#28 Tumors, Breast
#29 Breast Cancer
#30 #21 OR #22 OR #23 OR #24 OR #25 OR #26 OR #27 OR #28 OR #29
#31 MeSH descriptor: [Fatigue] explode all trees
#32 fatigue
#33 fatigue syndrome
#34 tiredness
#35 exhaustion
#36 energy depletion
#37 physical fatigue
#38 #31 OR #32 OR #33 OR #34 OR #35 OR #36 OR #37
#39 Randomized Controlled Trial
#40 RCT
#41 randomized trial
#42 controlled clinical trial
#43 randomized study
#44 randomized clinical trial
#45 RCTs
#46 randomized clinical studies
#47 intervention trial
#48 randomized intervention
#49 trial
#50 experimental study
#51 #39 OR #40 OR #41 OR #42 OR #43 OR #44 OR #45 OR #46 OR #47 OR #48 OR #49 OR #50
#52 #20 AND #30 AND #38 AND #51 in Trials

3. Encoding example

- **Agent:** The agent level.
- **METs_min:** The energy expenditure per min in the study.
- **Min_session：** The duration of a single exercise intervention.
- **Frequency:** The number of times participants engaged in physical activity (times/week).
- **Exact_dose:** The exact estimated METs accumulated per week by participants (METs-min/week).
- **Dose:** The dose group by approximation (METs-min/week).
- **CG:** Control group

Table S1. Dose categorization table

Study ID	Agent	METs_min	Min_session	Frequency	Prescribed dose	Average adherence rate (%)	Exact_dose	Dose
1	Yoga	2.3	60	5	690	70	483	500
1	CG	0	0	0	0	0	0	0

4. Table S2. Study characteristics

Study	Interventions (n)	Treatment types	Dose (METs-min/week)	Age (mean±sd)	Cancer Stage	Intervention length (weeks)	Baseline fatigue status	Fatigue Scale	Region	Exercise Attendance (%)	Exercise-related Adverse events (n)
Banasik et al. 2009 [1]	Yoga (9)	NA	500	63.33 (6.9)	II–IV	8	Non-severe	FACT-B	USA	87.5%	NA
	UC (9)		0	62.4 (7.3)							
Bower et al. 2011 [2]	Yoga (16)	NA	500	54.4 (5.7)	0-II	12	Severe	FSI	USA	78	Back spasm (1)
	UC (15)		0	53.3 (4.9)							
Calonego et al. 2023 [3]	AE-RT (10)	Surgery,	750	56.30 (9.91)	I - III	8	Non-severe	PFS	Brazil	99	NA
	AE-RT (9)	CPT, RDT	500	51.22 (12.57)							
Cantarero-Villanueva et	AE-RT (32)	NA	1000	49 (9)	I-IIIa	8	Severe	PFS	Spain	83.3	Neck-shoulder pain (3)
al. 2011 [4]	UC (35)		0	48 (9)							
Courneya et al.	AE (24)	Surgery,	1000	59 (5)	NA	15	Non-severe	FACT-F	Canada	98.4	NA
2002 [5]	UC (28)	CPT, RDT	0	58 (6)							
Cramer et al. 2015 [6]	Yoga (19)	Surgery,	250	48.3 (4.8)	I-III	12	Severe	FACIT-F	Germany	80.8	Sciatica (1)
	UC (21)	CPT, RDT	0	50.0 (6.7)							Pain (3)
Dieli-Conwright et al.	AE-RT (46)	CPT, RDT	1000	53.5 (10.4)	0-III	16	Severe	BFI	USA	96	NA
2018 [7]	UC (45)		0								
Erturhan Türk et al. 2024	AE (22)	CPT, RDT	250	45.86 (5)	II-III	12	Severe	PFS	Turkey	NP	NA
[8]	UC (23)		0	49 (5.38)							
Hagstrom et al. 2015 [9]	RT (20)	Surgery,	500	51.2 (8.5)	I-IIIa	16	Non-severe	FACIT-F	Australia	85	NA
	UC (19)	CPT, RDT	0	52.7 (8.8)							
Han et al. 2023 [10]	RT (23)	Surgery, CPT	500	49.91 (7.62)	I- II	12	Severe	R-PFS-K	Korea	NA	NA
	UC (23)		0	47.91 (6.41)							
Khan et al. 2020 [11]	AE (30)	Surgery,	250	NA	NA	6	Severe	FACIT-F	India	NA	NA

	RT (30)	CPT, RDT	250	NA							
Kiecolt-Glaser et al. 2014	Yoga (100)	Surgery,	250	51.8 (9.8)	0- IIIa	12	Severe	MFSI-SF	USA	97	Back pain (2)
[12]	UC (100)	CPT, RDT	0	51.3 (8.7)							
Kim et al. 2019 [13]	AE-RT (23)	Surgery, CPT	500	49.91 (7.62)	I–III	12	Severe	R-PFS-K	Korea	NA	NA
	UC (25)		0	48.48 (6.75)							
Littman et al. 2012 [14]	Yoga (32)	NA	500	60.6 (7.1)	0–III	24	Non-severe	FACIT-F	USA	87	NA
	UC (31)		0	58.2 (8.8)							
Luca et al. 2016 [15]	AE-RT (10)	NA	1000	50.2 (9.7)	I -III	24	Non-severe	FACIT-F	Italy	NA	NA
	UC (10)		0	46 (2.8)							
Milne et al. 2008 [16]	AE-RT (29)	NA	1250	55.2 (8.4)	I–II	12	Severe	SCFS	Australia	60.4	NA
	UC (29)		0	55.1 (8)							
Moraes et al. 2021 [17]	RT (13)	Surgery,	250	55 (5.8)	NA	8	Severe	PFS	Brazil	NA	NA
	UC (13)	CPT, RDT	0	54.3 (5.2)							
Nouri et al. 2018 [18]	RT (25)	Surgery,	250	46 (5.8)	0–III	6	Non-severe	Pipper	Iran	NA	NA
	UC (25)	CPT, RDT	0	46 (7.2)							
Nyrop et al. 2017 [19]	AE (31)	NA	250	63.3 (6.9)	NA	6	Non-severe	VAS	USA	NA	NA
	UC (31)		0	64.4 (9.7)							
Ochi et al. 2022 [20]	AE-RT (21)	NA	500	NA	I–IIa	12	Non-severe	CFS	Japan	86	NA
	UC (23)		0	NA							
Pagola et al. 2020 [21]	AE-RT (13)	CPT, RDT	1000	47 (7)	NA	16	Severe	PERFORM	Spain	83	NA
	AE-RT (10)		1250	51 (6)							
Pinto et al. 2015 [22]	AE (39)	Surgery,	750	55.64 (8.59)	0 -III	12	Non-severe	FACIT-F	UK	NA	NA
	UC (37)	CPT, RDT	0	55.59 (10.59)							
Qiao et al. 2022 [23]	AE (22)	Surgery,	750	55.9 (8.2)	NA	8	Non-severe	BFI	USA	74	NA
	UC (19)	CPT, RDT	0	53.8 (11.4)							Related and nonserious.
Rogers et al. 2012 [24]	AE-RT (15)	Surgery,	1000	58.0 (6.1)	I-IIIa	12	Non-severe	FSI	USA	100	events (2)

	UC (13)	CPT, RDT	0	53.7 (13.9)						
Rogers et al. 2014 [25]	AE-RT (20)	Surgery,	1000	57.2 (5.5)	0 -II	12	Severe	FSI	USA	NA	NA
	UC (24)	CPT, RDT	0	55.2 (9.1)						
Saarto et al. 2012 [26]	AE (263)	Surgery,	1000	61.7 (9.4)	I–III	48	Non-severe	CFS	Finland	62	NA
	UC (237)	CPT, RDT	0	59.3 (11)						
Schad et al. 2023 [27]	AE (30)	Surgery,	500	61.7 (9.4)	I–III	6	Severe	CFS	Argentina	89	NA
	UC (30)	CPT, RDT	0	59.3 (11)						
Shobeiri et al. 2016 [28]	AE (30)	Surgery,	500	42.7 (9.6)	I-II	10	Severe	QLQ-C30	Iran	NA	NA
	UC (30)	CPT, RDT	0	43.5 (8.6)						
Soriano-Maldonado	RT (32)	Surgery,	500	52.6 (8.8)	NA	12	Non-severe	FACT-F	Spain	75	NA
et al. 2022 [29]	UC (28)	CPT, RDT	500	52.0 (9.4)						
Stan et al. 2016 [30]	RT (16)	Surgery,	250	63.0 (9.3)	0 -II	12	Severe	MFSI-SF	USA	68	NA
	Yoga (18)	CPT, RDT	250	61.4 (7.0)						
Vardar et al. 2015 [31]	Yoga (19)	NA	500	49.89 (4.65)	NA	6	Severe	FSS	Turkey	NA	NA
	UC (21)		0	47.38 (7.57)						
Yuen et al. 2007 [32]	AE (8)	CPT, RDT	250	53.1 (13.5)	NA	12	Non-severe	PFS	USA	76.2	NA
	RT (7)		250	53.7 (11.3)						
	UC (7)		0	55 (13.4)						

Note: NA: Not Available; CPT: Chemothera Peutic Treatment；RDT: Radiotherapeutic Treatment; UC: Usual Care; AE: Aerobic Exercise; AE-RT: Combined Aerobic and Resistance Training; RT: Resistance Training; FACIT-F: Functional Assessment of Chronic Illness Therapy – Fatigue; PFS: Piper Fatigue Scale; FACT-B: Functional Assessment of Cancer Therapy-Breast; FACT-F: Functional Assessment of Cancer Therapy – Fatigue instrument; FSI: Fatigue Symptom Inventory; BFI: Brief Fatigue Inventory; QoLC30: European Organization for the Research and Treatment of Cancer—Quality of Life; FSS: Fatigue severity scale; PERFORM: Psychometric properties of the Perform Questionnaire; CFS: Cancer Fatigue Scale；R-PFS-K: Korean Revised Piper Fatigue Scale; MFSI-SF: Multidimensional Fatigue Symptom Inventory-Short Form; VAS: Visual Analogue Scale; SRF: Self-Reported Fatigue

5. Risk of bias

Risk of bias was assessed using the following criteria. For the randomization process, although all RCTs employed random allocation, those not reporting specific methods (e.g., computer-generated) were rated as "some concerns." Due to the nature of exercise interventions, participant blinding was generally not feasible; therefore, this domain was conservatively rated as "some concerns." In addition, studies that did not clearly report blinding of personnel responsible for allocation were considered to have a high risk of bias. For studies with missing outcome data, the risk of bias was rated as "some concerns" (<90%) or "high risk" (<80%). However, if appropriate statistical methods were used to handle missing data (e.g., intention-to-treat analysis or multiple imputation), the study was rated as low risk. Regarding the measurement of the outcome, although validated and reliable instruments were used across studies, the subjective nature of these assessments may be influenced by the lack of blinding, potentially leading to social desirability or expectation bias. Therefore, this domain was consistently rated as "some concerns." Regarding selective reporting, most studies were preregistered and followed predefined protocols. Studies without a registration number were considered at high risk, as the reporting bias could not be fully assessed. If a study had "some concerns" or "high risk" in any domain, the overall risk was rated as "some concerns" or "high risk."

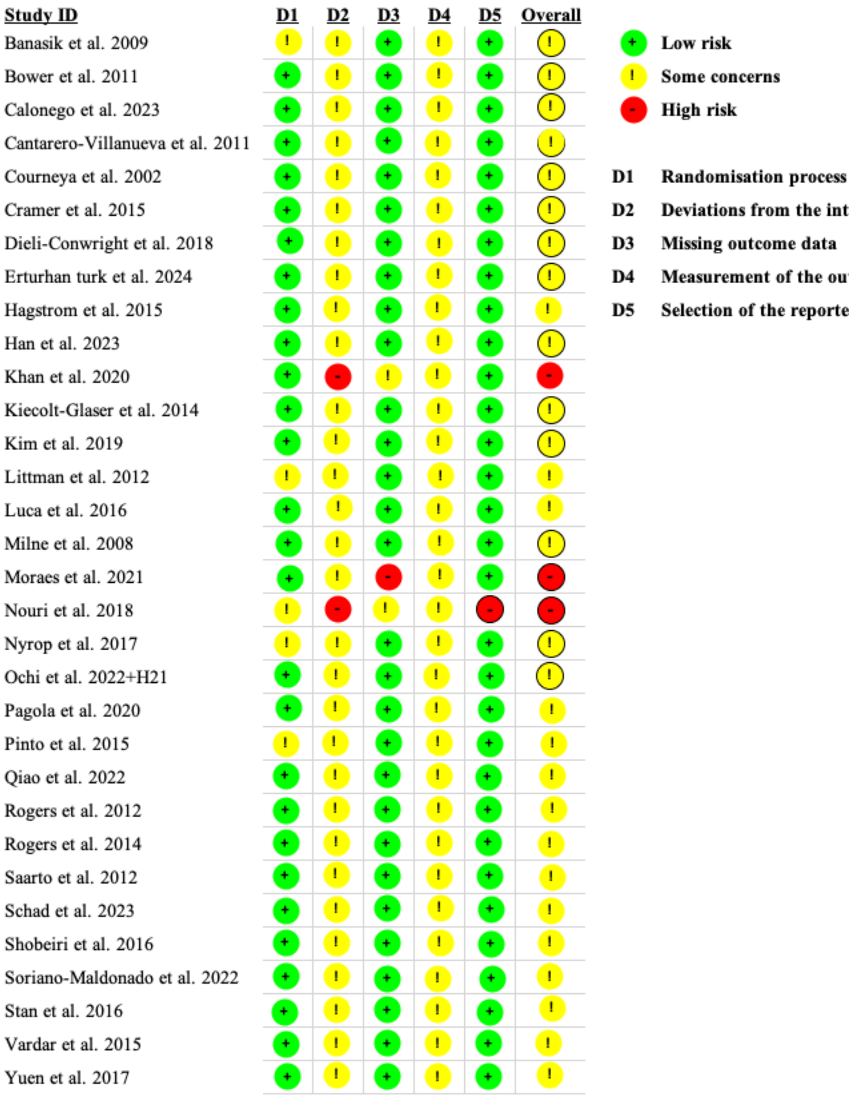

Figure S1. The detail of risk of bias.

6. Dose-response network meta-analyses

6.1 Connectivity

Connectivity is a key assumption in network meta-analysis (NMA) that, if considered insufficient (i.e., due to lack of direct comparisons), may lead to low statistical power and misleading results. Our study assessed network connectivity at the motor and dose levels and did not find any evidence of network unconnectedness (Figure 3; Figure S2).

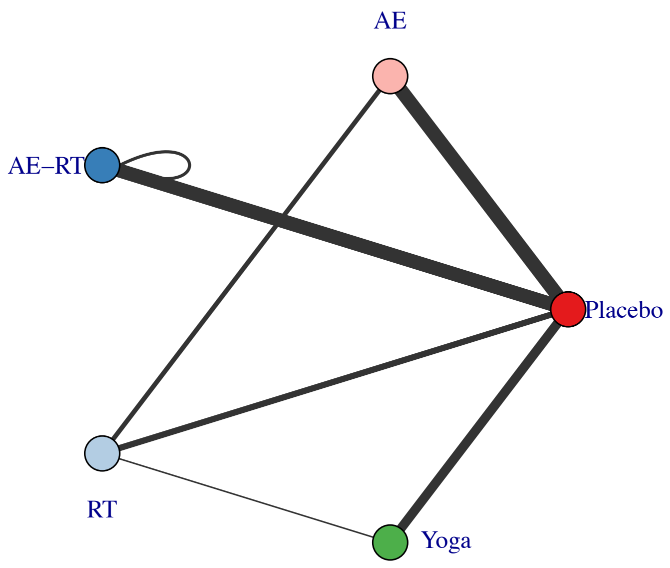

Figure S2. Agent-level network.

Line thickness represents the number of studies available for comparison. AE: Aerobic Exercise; AE-RT: Combined Aerobic and Resistance Training; RT: Resistance Training

6.2 Consistency

We performed a consistency analysis of the data by comparing the consistency of the network (i.e., network effect size) with the unrelated mean effects (UME) model (i.e., pairwise effect sizes). In practice, we checked whether the bias, the number of estimated parameters in the network, and the Deviance Information Criterion (DIC) metric were similar for both models, indicating a good fit. The comparison of these parameters showed a good agreement between the different models (Table S3).

Table S3. Consistent and UME models fit comparison.

Model	pD	Deviance	Residual deviance	DIC	SD
Consistent	58.1	187.8	102.89	220.6	3.92
UME	59	188	103	221.9	4

Note: UME = unrelated mean effects model; DIC = deviance information criterion; pD = effective number of parameters; SD = standard deviation

6.3 Transitivity

NMAs are based on the assumption of indirect/mixed comparisons, which implies that estimates of treatment effects from direct and indirect evidence are consistent but with the usual variation of meta-analyses under a random effects model. This assumption is equivalent to heterogeneity in a "standard" meta-analysis. Following a previous proposal, anomalies were assessed at a deeper network level (i.e., at the treatment level). We assessed the span by the MBNMA node-splitting method. This method splits the contribution of a specific treatment contrast into direct and indirect evidence and compares them. Similar effects indicate good span. **Table S4** presents the results for transitivity in this meta-analysis.

Comparison	p-value	Median	2.50%	97.50%
Yoga_250 vs RT_250	0.338			
-> direct		4.665	-4.708	14.177
-> indirect		-0.305	-3.499	2.84
-> MBNMA		0.24	-2.66	3.37
AE-RT_1250 vs AE-RT_1000	0.061			
-> direct		-0.036	-5.672	5.699
-> indirect		0.033	0.001	0.126
-> MBNMA		0.031	0.001	0.128
AE-RT_750 vs AE-RT_500	0.65			
-> direct		1.643	-4.34	7.47
-> indirect		0.096	0.003	0.317
-> MBNMA		0.091	0.003	0.335
RT_500 vs AE_250	0.519			
-> direct		0.293	-5.586	6.3
-> indirect		-2.09	-5.2	0.86
-> MBNMA		-1.83	-4.46	0.86
RT_250 vs AE_250	0.243			
-> direct		4.13	0.82	7.52
-> indirect		0.46	-2.54	3.26
-> MBNMA		-1.4	-3.71	1.05
Yoga_500 vs Placebo_0	0.243			
-> direct		4.126	0.822	7.521
-> indirect		0.46	-2.54	3.285
-> MBNMA		-1.397	-3.706	1.05
AE-RT_500 vs Placebo_0	0.608			
-> direct		1.41	-1.9	4.87
-> indirect		2.75	0.55	5.31
-> MBNMA		2.344	0.6	4.28
AE_1000 vs Placebo_0	0.681			
-> direct		1.395	-2.818	5.421
-> indirect		2.546	0.03	5.44

-> MBNMA		2.12	0.09	4.37
AE_750 vs Placebo_0	0.686			
-> direct		2.451	-2.416	7.23
-> indirect		2.12	-0.18	4.89
-> MBNMA		2.07	0.09	4.24
AE_500 vs Placebo_0	0.079			
-> direct		10.905	5.374	17.001
-> indirect		0.944	-0.93	2.798
-> MBNMA		1.966	0.086	3.966

Note: AE: Aerobic Exercise; AE-RT: Aerobic combined with Resistance Training; RT: Resistance Training

6.4 Models' selection

6.4.1 Linear and non-linear functions and models fit comparison

A meta-analysis—specifically, a "split" network meta-analysis (NMA)—was conducted by treating different doses of physical activity as separate and unrelated interventions. This step aimed to explore the dose–response pattern and assess which functional form best fits the data, thereby informing the choice of model for the subsequent model-based network meta-analysis (MBNMA) [33]. The results of the split NMA are presented in Figure S3 and Figure S4, showing the SMD in fatigue across various exercise doses overall and by exercise type, respectively.

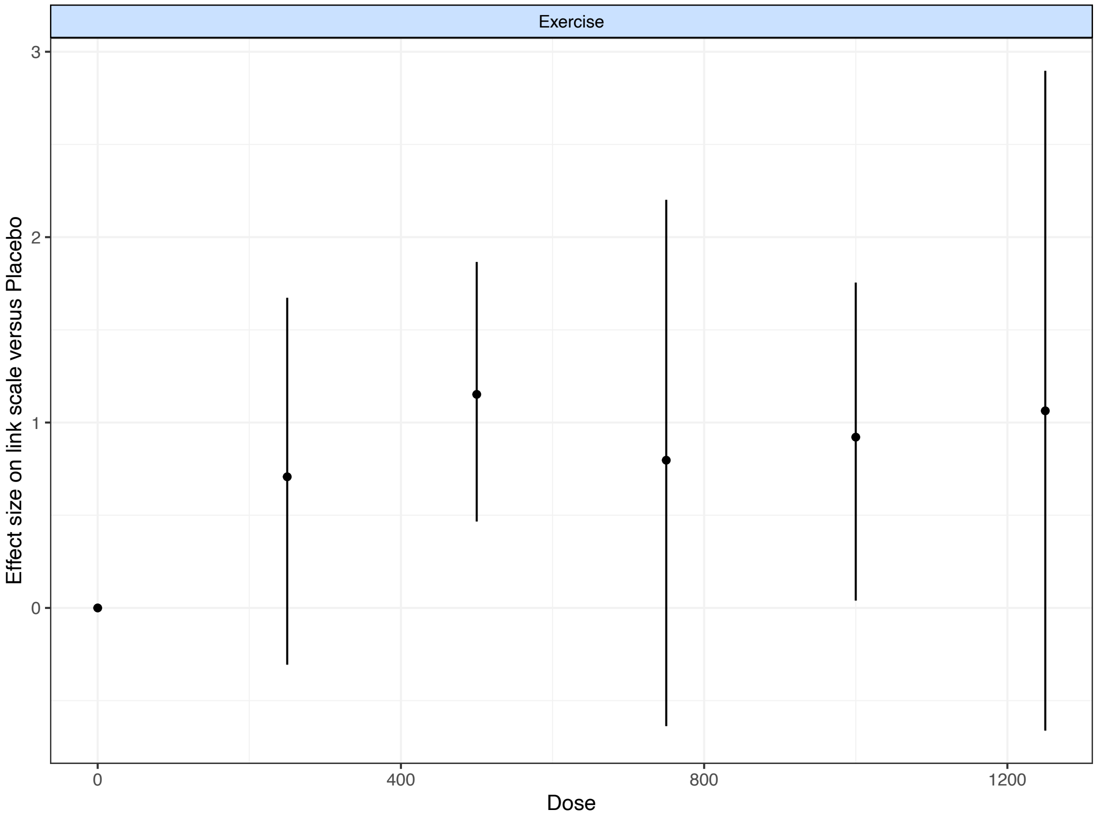

Figure S3. "Split" NMA of overall exercise.

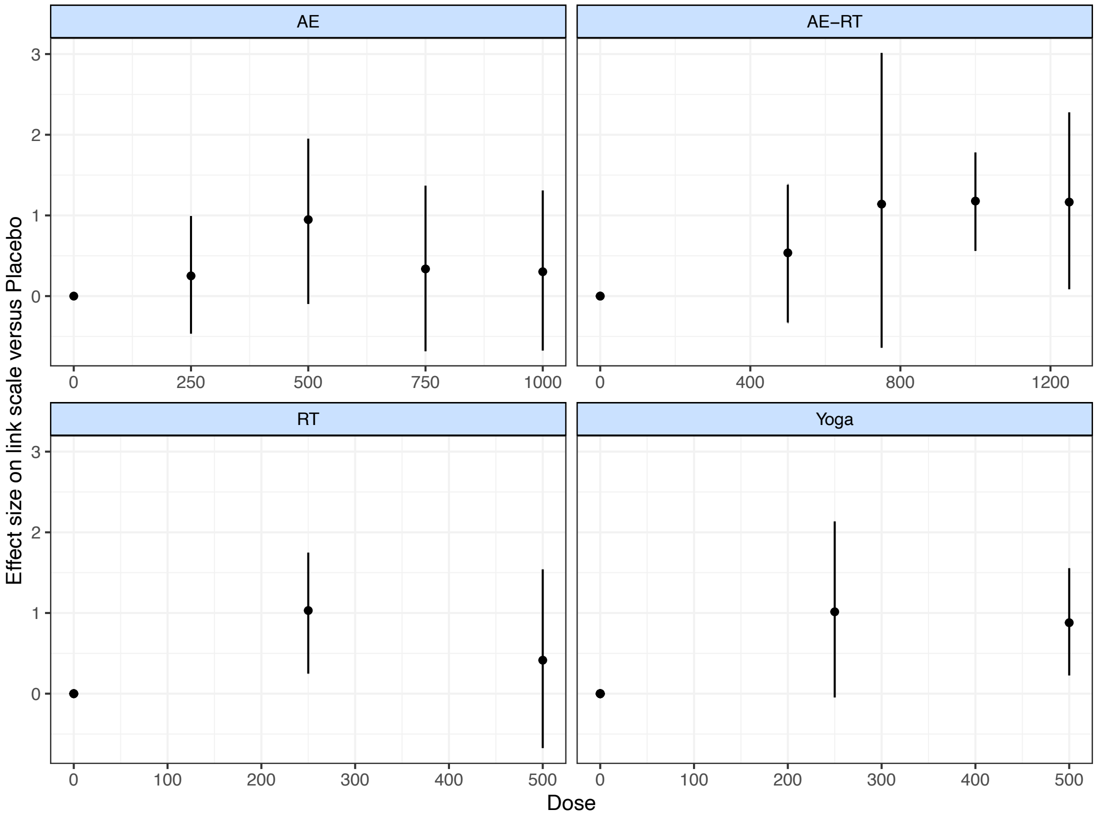

Figure S4. “Split” NMA of different exercise agents.

AE: Aerobic Exercise; AE-RT: Aerobic combined with Resistance Training; RT: Resistance Training

6.4.2. Models fit comparison

In addition to model fit indices, deviation plots help assess model robustness by showing each data point's contribution to the posterior mean deviance, with values near 1 indicating good fit [34]. All points were close to 1, suggesting an overall good model fit. Deviation plots for overall (Figure S5) and treatment-level plots (Figure S6) confirm the robustness of our model selection.

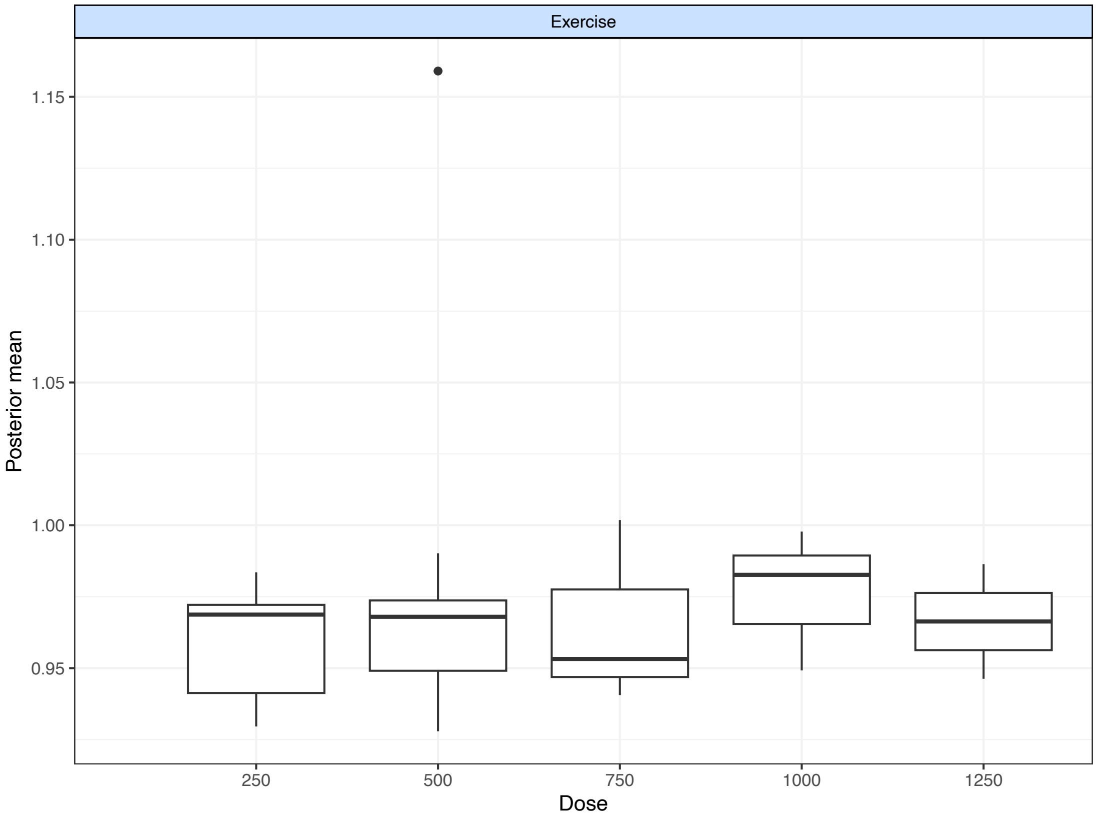

Figure S5. Deviance plot at overall exercise.

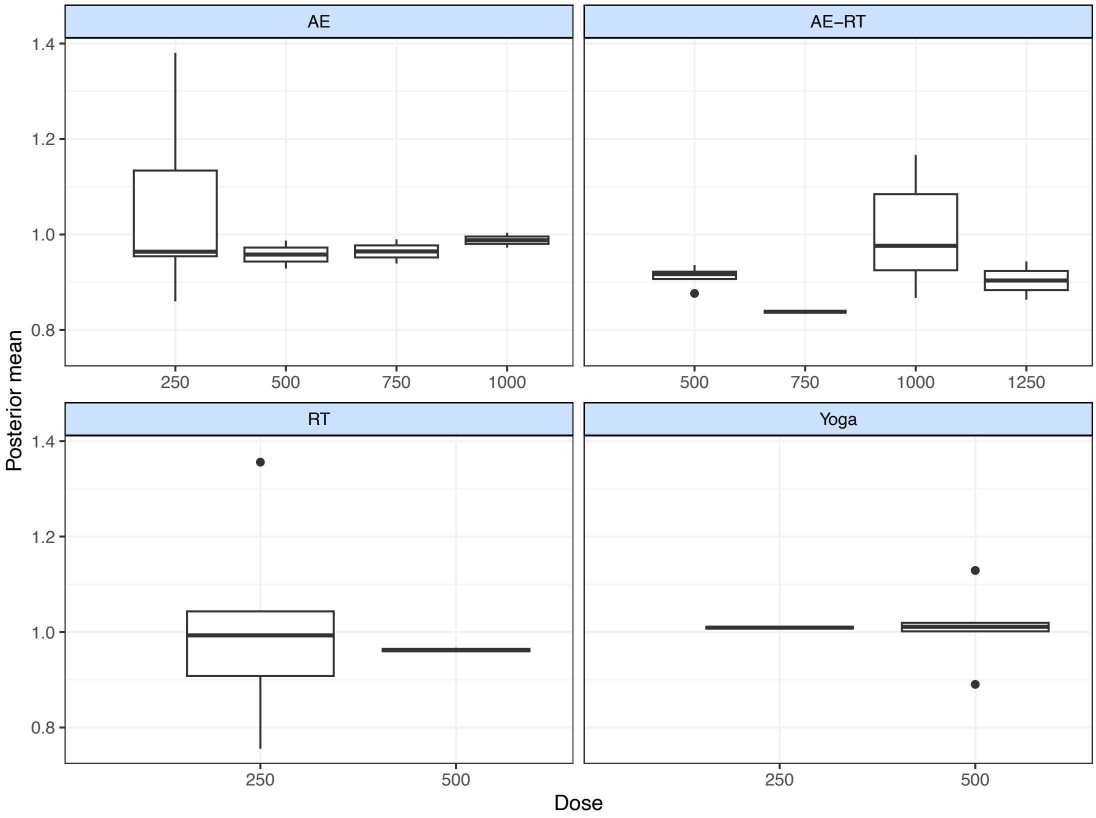

Figure S6. Deviance plots at treatment-level.

AE: Aerobic Exercise; AE-RT: Aerobic combined with Resistance Training; RT: Resistance Training

Additionally, we plotted the model fit to assess its degree of accuracy. The fit values are represented as connecting lines, while the original dataset observations are shown as points. These plots help evaluate the model's fit across different exercise types and doses in the dose-response function (Figure S7, S8).

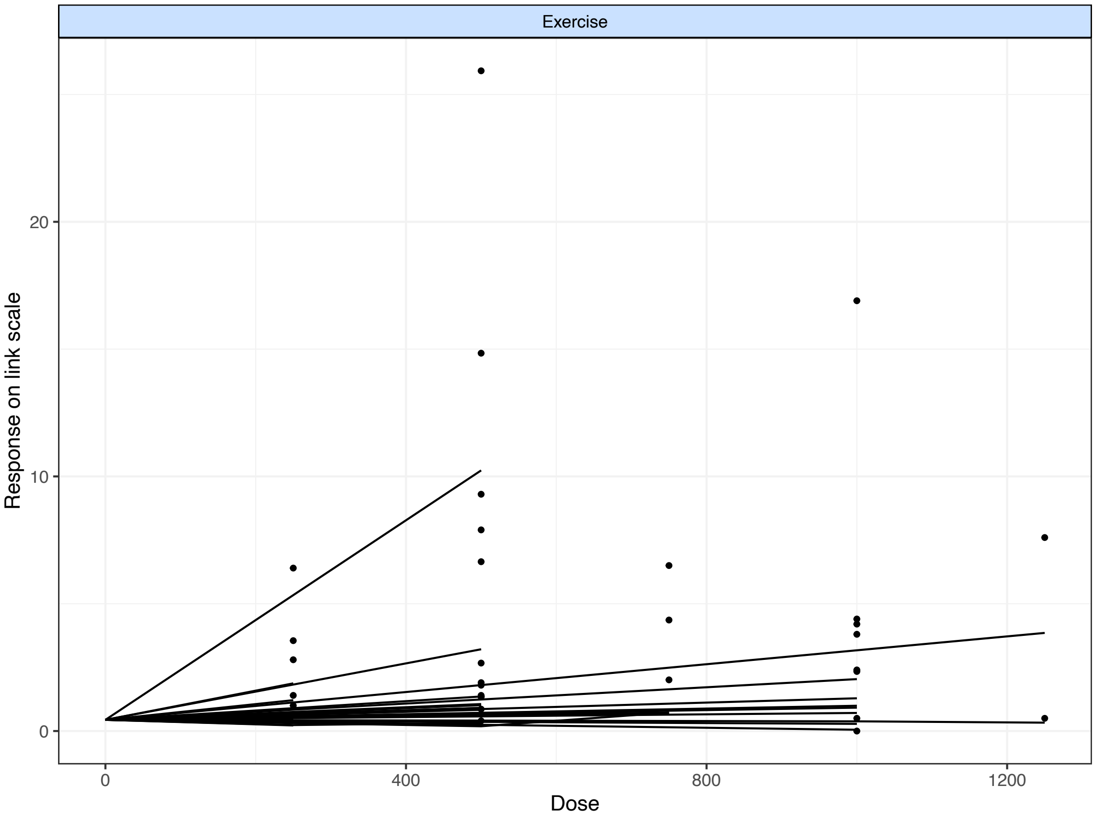

Figure S7. Fit plots at overall exercise level.

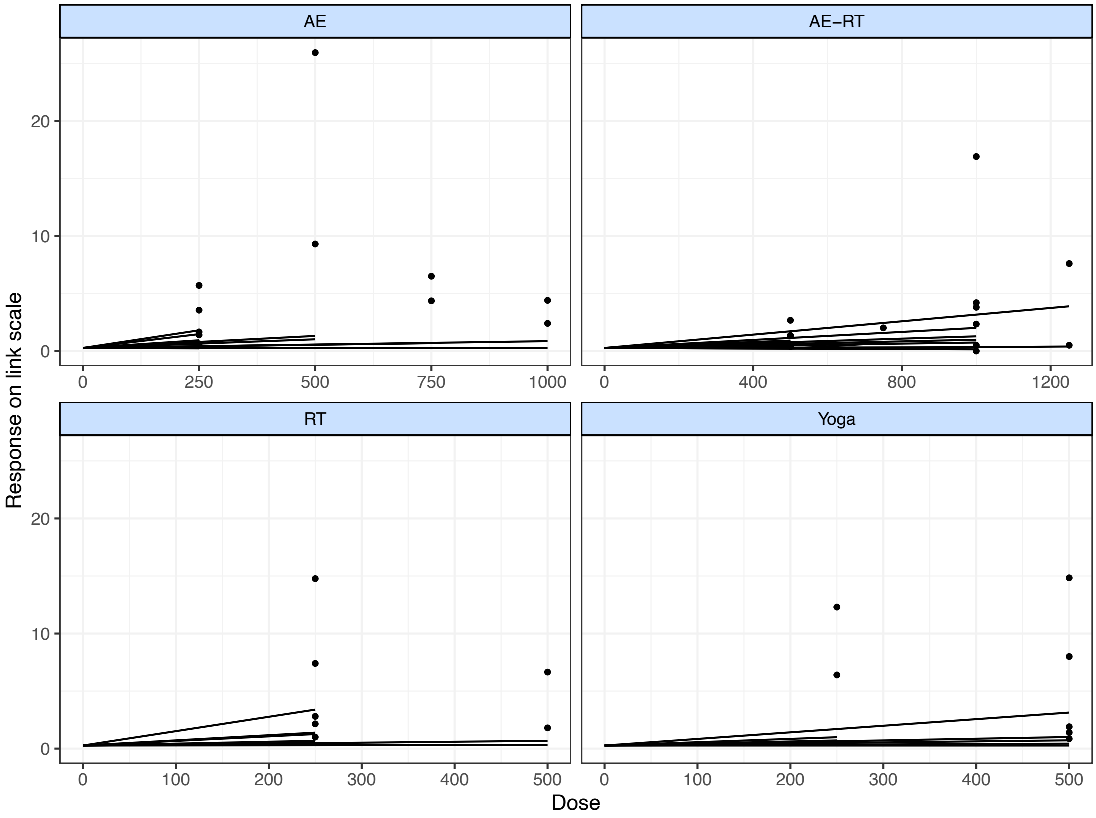

Figure S8. Fit plots at agent-level.

AE: Aerobic Exercise; AE-RT: Aerobic combined with Resistance Training; RT: Resistance Training

6.4.3 Table S5. Models fit comparison

There are two reasons for selecting the quadratic function: 1) It results in the lowest DLC; 2) We compared different models and found that the quadratic function better aligns with the dose-response curves shown in Figures S3 and S4.

Model	DIC	SD	Deviance	Residual deviance	pD
Emax (random treatment effects)	246	1.023	168.04	79.22	78
Restricted cubic spline (random treatment effects; 3 knots)	247.6	1.125	168.34	79.53	79.1
Log Linear (random treatment effect)	244.8	1.027	167.93	79.12	77.1
Non-parametric monotonically up	246.4	1.129	168.99	80.17	78.2

(random treatment effects)					
Exponential dose-response function (random treatment effects)	244.2	1.028	168.15	79.33	76.8
Quadratic function (random treatment effects)	243.8	1.05	168.61	79.8	78.3
Spline dose-response functions (random treatment effects)	248	1.13	169.02	80.206	79.2
Fractional polynomial dose-response function (random treatment effects)	246.1	1.097	167.48	78.67	78

Note: DIC = deviance information criterion; pD = effective number of parameters; SD = standard deviation

7. Dose-response relationships

Considering the uneven distribution of data across dose levels, the observed decline in effects may be driven by data sparsity rather than reflecting a true reduction in efficacy.

7.1 Dose-response relationship between exercise dose and fatigue

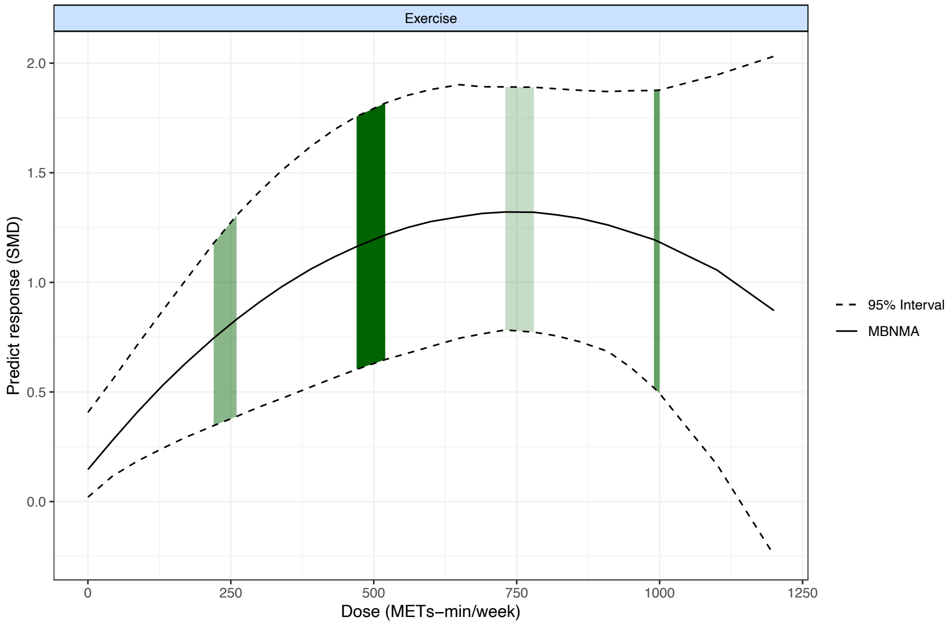

Figure S9. Dose-response relationship between overall exercise dose and fatigue in breast cancer survivors (the green area represents the original study dataset; the darker the color, the larger the amount of data).

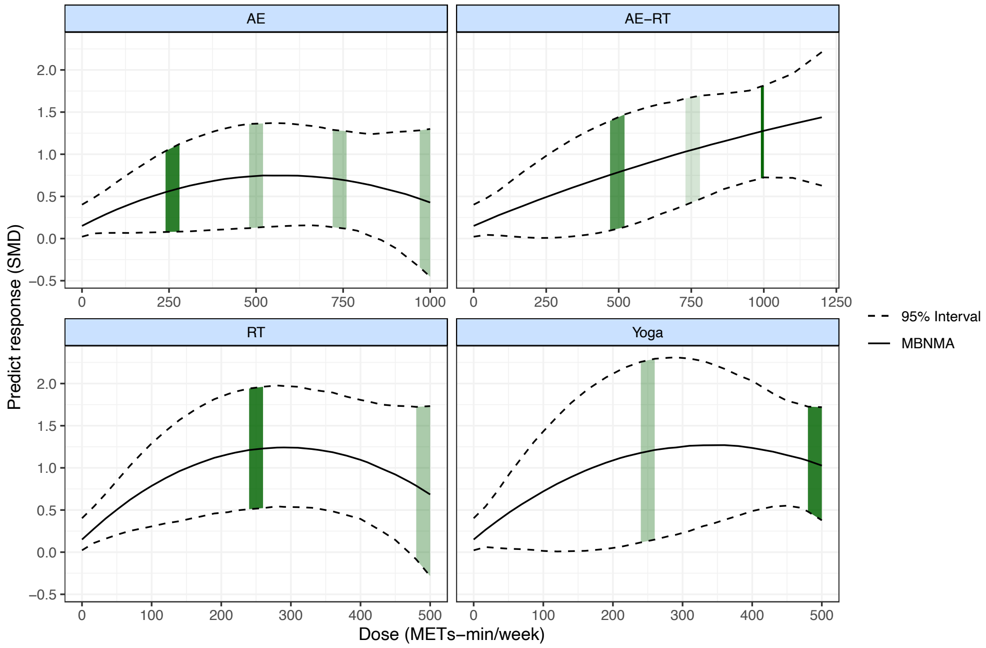

Figure S10. Dose-response relationship between different exercise dose and fatigue in breast cancer survivors (the green area represents the original study dataset; the darker the color, the larger the amount of data). AE: Aerobic Exercise; AE-RT: Aerobic combined with Resistance Training; RT: Resistance Training

7.2 Effectiveness ranking of different exercises and doses

Table S6. Predictions ranking of different exercise (from best to worst).

Rank	Treatment	Mean	Median	2.50%	97.50%
1	AE-RT_1200	18.8696666666667	10	1	85
2	AE-RT_1100	19.4923333333333	14	2	69
3	Yoga_330	24.9326666666667	17	2	83.0250000000001
4	Yoga_340	24.747	17	2	81.0250000000001
5	Yoga_310	25.793	18	1	88
6	Yoga_360	24.88	18	2	77
7	AE-RT_1000	22.0436666666667	19	3	64
8	Yoga_290	27.4096666666667	19	1	91
9	AE-RT_990	22.9663333333333	20	4	64
10	Yoga_380	25.4846666666667	20	3	74
11	RT_280	24.0786666666667	21	2	67
12	Yoga_280	28.8936666666667	21	1	94
13	RT_290	23.983	21.5	2	67
14	AE-RT_950	24.924	22	4	66
15	RT_260	25.1256666666667	22	1	69
16	Yoga_400	26.5516666666667	22	4	71
17	RT_310	24.6436666666667	23	2	65
18	RT_240	27.2296666666667	24	2	71.0250000000001
19	RT_330	26.1746666666667	24	2	67
20	Yoga_260	31.681	24	1	96
21	Yoga_410	27.487	24	5	70
22	AE-RT_910	27.217	25	4	68
23	RT_340	27.4656666666667	26	3	67
24	RT_220	30.2723333333333	27	3.975	75
25	Yoga_430	29.5613333333333	27	5	70
26	AE-RT_860	30.234	28	3	73
27	Yoga_240	34.905	28	4	99.0250000000001
28	RT_360	30.2376666666667	29	4	71
29	RT_210	32.502	30	6	77
30	Yoga_450	32.3806666666667	30	4	76
31	AE-RT_820	33.1223333333333	31	3	76.0250000000001
32	RT_380	33.7013333333333	32	5	78
33	Yoga_220	38.4003333333333	32	6	101
34	RT_190	36.465	34	8	80
35	Yoga_210	40.7396666666667	34	8	103
36	Yoga_470	35.8833333333333	34	3	82
37	AE-RT_780	36.2316666666667	35	3	80
38	RT_400	37.9796666666667	37	6	86
39	Yoga_480	38.255	37	2	88
40	RT_170	41.0053333333333	39	11	84
41	AE-RT_730	40.1503333333333	40	4	85

42	RT_410	40.8736666666667	40	6	90.025	
43	Yoga_190	44.631	40	10		105
44	RT_160	44.038	42	13	86.0250	
45	Yoga_500	43.1046666666667	42	1		98
46	AE-RT_690	43.655	43	5		89
47	RT_430	46.5216666666667	45	5		100
48	Yoga_170	48.9713333333333	45	12		107
49	AE-RT_650	47.2473333333333	47	7		93
50	RT_140	49.6116666666667	48	16		90
51	Yoga_160	51.8326666666667	48	14		108
52	AE-RT_600	51.6183333333333	52	9		96
53	RT_450	52.865	52	4		110
54	Yoga_140	57.023	54	17		109
55	RT_120	56.0596666666667	55	22		94
56	AE-RT_560	55.374	56	12		99
57	RT_470	59.698	59	3		112
58	AE-RT_520	59.1603333333333	60	14		102
59	Yoga_120	62.898	61	20		108
60	RT_100	63.456	63	30.975		97
61	RT_480	63.5436666666667	63	2		113
62	AE_520	62.4253333333333	64	14		99
63	AE_550	62.007	64	14		98
64	AE-RT_470	63.711	64	16		104
65	AE_590	62.1846666666667	65	15		97
66	AE_620	62.801	65	17		97
67	AE_480	63.6446666666667	66	15		99
68	AE_660	64.0703333333333	67	19		97
69	AE_450	65.3313333333333	68	17		101
70	AE_690	65.4483333333333	68	21		100
71	AE-RT_430	67.699	68	17		106
72	RT_86	69.4473333333333	69	36.975		100
73	Yoga_100	69.4423333333333	69	26		109
74	AE_410	68.011	70	21		102
75	AE_720	67.0656666666667	70	21		102
76	AE_760	69.411	72	23		106
77	AE_380	70.5623333333333	73	24		103
78	AE-RT_390	71.7383333333333	73	22		108
79	RT_500	70.4263333333333	73	1		114
80	AE_790	71.5273333333333	74	24.975		108
81	AE_340	73.9806666666667	76	29		105
82	Yoga_86	74.7653333333333	76	34		109
83	AE_830	74.318	77	25		109
84	RT_69	77.037	78	47		103
85	AE_310	76.956	79	33		106

86	AE_860	76.6586666666667	79	23	110
87	AE-RT_340	76.65	80	30	110
88	AE_280	80.072	82	39	107
89	AE_900	79.6513333333333	83	21	111
90	Yoga_69	81.4996666666667	84	45	110
91	AE-RT_300	80.864	85	36	111
92	AE_240	84.032	86	46.975	108.025
93	AE_930	81.9953333333333	87	19	112
94	RT_52	85.2386666666667	87	56	106
95	AE_210	87.3186666666667	89	53	109
96	AE-RT_260	85.1723333333333	89	42	112
97	AE_970	84.6776666666667	92	16.975	113
98	Yoga_52	88.6616666666667	92	56	110
99	AE_170	91.5	93	61	109
100	AE-RT_220	89.3846666666667	93	49	112
101	AE_1000	86.709	96	13	114
102	RT_34	93.985	96	68	109
103	AE_140	94.9523333333333	97	68	110
104	AE-RT_170	94.5083333333333	98.5	59	112
105	Yoga_34	96.441	99	69	111
106	AE_100	99.2833333333333	101	76	111
107	AE-RT_130	98.7416666666667	102	68	112
108	AE_69	102.93	105	81	112
109	RT_17	102.522333333333	105	79	112
110	AE-RT_86	103.113	106	76	112
111	Yoga_17	103.848666666667	106	81	113
112	AE_34	106.973	109	87	113
113	AE-RT_43	107.139333333333	110	84	113
114	Placebo_0	110.180666666667	113	90	114

Note: The number that follows the exercise intervention indicates the dose of exercise (METs-min/week). AE: Aerobic Exercise; AE-RT: Combined Aerobic and Resistance Training; RT: Resistance Training

8. Publication bias

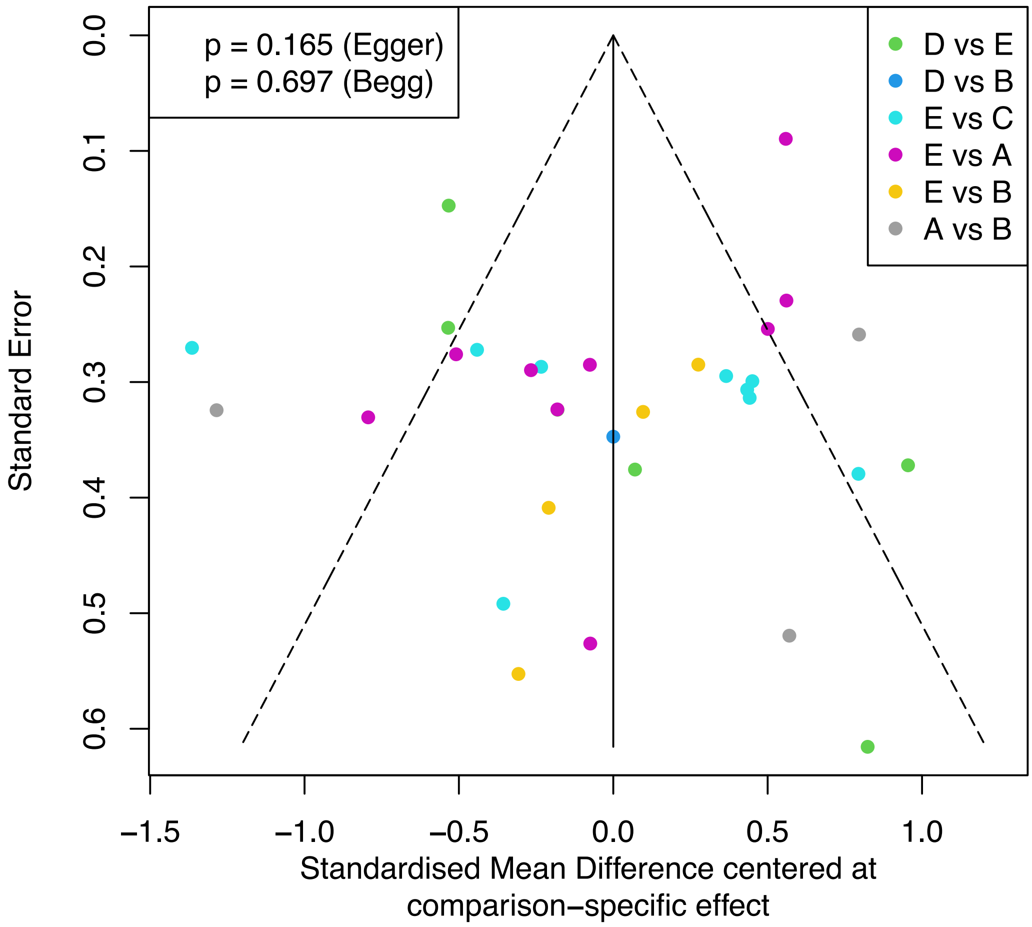

Figure S11. Funnel plot.

A: Aerobic Exercise; B: Resistance Training; C: Combined Aerobic and Resistance Training; D: Yoga; E: Control group

9. Grade summary of all studies

Based on the CINeMA online guidelines (https://cinema.ispm.unibe.ch/), we evaluated only the fatigue results. Downgrading decisions were made based on the following criteria:

9.1 Within-study Bias

Each included study was rated for risk of bias as low (RoB2 1 point), moderate (RoB2 2 points), or high (RoB2 3 points). The rule applied was the average RoB. No downgrade occurred for "no concerns"; one level was downgraded for "some concerns," and two levels for "major concerns."

9.2 Reporting Bias (Publication Bias and Selective Reporting)

To minimize publication bias, we examined the reference lists of recent relevant studies. Publication bias was assessed using comparison-adjusted funnel plots, Begger and Egger's test (Supplementary Content 8). The results indicated no statistically significant bias ($p > 0.05$), suggesting a low risk of publication bias. Furthermore, only one studies were rated as high risk for selective reporting; therefore, this domain was not downgraded.

9.3 Indirectness

Indirectness was assessed by examining the clinical and methodological relevance of the studies to the research question. All studies met the PICOS criteria, targeting adults with breast cancer survivors, comparing different exercise interventions to appropriate controls, and reporting fatigue as the outcome. Since all studies were directly relevant, no concerns about indirectness were identified, and no downgrading occurred in this area.

9.4 Imprecision

Fatigue, the outcome of this network meta-analysis, was treated as a continuous variable. Standardized mean difference (SMD) was used to measure effect size (change score: endpoint minus baseline score) due to variations in rating scales or units. A clinically meaningful effect size was considered if the SMD was higher or lower than 0.2 (small effect size). Downgrading occurred if the confidence interval crossed one threshold (one level) or two thresholds (two levels).

9.5 Heterogeneity

Heterogeneity was judged based on both statistical and clinical considerations. Given that the included studies met the PICOS criteria and focused on breast cancer survivors after primary treatment, and that heterogeneity across exercise types and doses was explicitly considered, no downgrading was applied for clinical heterogeneity. However, all interventions were downgraded by at least one level due to statistical heterogeneity.

9.6 Incoherence

Incoherence was addressed by p-value thresholds: no downgrade for $p > 0.1$, one level for p 0.05–0.1, and two levels for $p < 0.05$.

9.7 Summarizing Judgments Across CINeMA Domains

The CINeMA framework assesses confidence in evidence across six domains: within-study bias, reporting bias, indirectness, imprecision, heterogeneity, and incoherence. These judgments are summarized into an overall confidence rating using the GRADE approach (high, moderate, low, or very low). As a single issue may impact multiple domains (e.g., indirectness due to intransitivity or heterogeneity affecting both imprecision and bias), all domains are considered together to avoid redundant downgrading and ensure a balanced assessment.

Table S7. The confidence of evidence using the CINeMA.

Comparison	Within-study bias	Reporting bias	Indirectness	Imprecision	Heterogeneity	Incoherence	Confidence rating	Reason(s) for downgrading
**AE:CG**	Some concerns	Low risk	No concerns	No concerns	Some concerns	Some concerns	**Very Low**	["Heterogeneity", Incoherence, Within-study bias]
**AE-RT:CG**	Some concerns	Low risk	No concerns	No concerns	Some concerns	No concerns	**Low**	["Heterogeneity", Within-study bias]
**RT:CG**	Some concerns	Low risk	No concerns	No concerns	Some concerns	No concerns	**Low**	["Within-study bias","Heterogeneity"]
**Yoga: CG**	Some concerns	Low risk	No concerns	No concerns	Some concerns	No concerns	**Low**	["Within-study bias", "Heterogeneity"]

Note: AE: Aerobic Exercise; AE-RT: Combined Aerobic and Resistance Training; RT: Resistance Training; CG: Control Group

References

1. Banasik J, Williams H, Haberman M, Blank SE, Bendel R. Effect of Iyengar yoga practice on fatigue and diurnal salivary cortisol concentration in breast cancer survivors. Journal of the American Association of Nurse Practitioners. 2011;23(3):135-42.
2. Bower JE, Garet D, Sternlieb B, Ganz PA, Irwin MR, Olmstead R, et al. Yoga for persistent fatigue in breast cancer survivors: a randomized controlled trial. Cancer. 2012;118(15):3766-75.
3. Calonego C, Alberton CL, Santagnello SB, Schaun GZ, Petrarca CR, Umpierre D, et al. Impact of resistance training volume on physical and perceptual outcomes of breast cancer survivors submitted to a combined training program: a randomized, single-blinded study. Journal of Physical Activity and Health. 2023;20(3):204-16.
4. Cantarero-Villanueva I, Fernández-Lao C, Díaz-Rodriguez L, Fernández-de-las-Peñas C, del Moral-Avila R, Arroyo-Morales M. A multimodal exercise program and multimedia support reduce cancer-related fatigue in breast cancer survivors: a randomised controlled clinical trial. European Journal of Integrative Medicine. 2011;3(3):e189-e200.
5. Courneya KS, Mackey JR, Bell GJ, Jones LW, Field CJ, Fairey AS. Randomized controlled trial of exercise training in postmenopausal breast cancer survivors: cardiopulmonary and quality of life outcomes. Journal of clinical oncology. 2003;21(9):1660-8.
6. Cramer H, Rabsilber S, Lauche R, Kümmel S, Dobos G. Yoga and meditation for menopausal symptoms in breast cancer survivors—a randomized controlled trial. Cancer. 2015;121(13):2175-84.
7. Dieli-Conwright CM, Courneya KS, Demark-Wahnefried W, Sami N, Lee K, Sweeney FC, et al. Aerobic and resistance exercise improves physical fitness, bone health, and quality of life in overweight and obese breast cancer survivors: a randomized controlled trial. Breast cancer research. 2018;20(1):124.
8. Türk KE, Yılmaz M. Efficacy of a Home-Based, Unsupervised Physical Activity Program on Fatigue, Sleep Quality, and Quality of Life in Survivors of Breast Cancer. Clinical journal of oncology nursing. 2024;28(1).
9. Hagstrom AD, Marshall PW, Lonsdale C, Cheema BS, Fiatarone Singh MA, Green S. Resistance training improves fatigue and quality of life in previously sedentary breast cancer survivors: a randomised controlled trial. European journal of cancer care. 2016;25(5):784-94.
10. Han J, Jang MK, Lee H, Kim SY, Kim SH, Hee Ko Y, et al. Long term effects of a social capital-based exercise adherence intervention for breast cancer survivors with moderate fatigue: A randomized controlled trial. Integrative cancer therapies. 2023;22:15347354231209440.
11. Khan S, Agrawal R, Shaikh S, Thakur N. Comparison of effect of aerobic training versus resistance training on cancer-related fatigue and quality of life in breast cancer survivors. Indian Journal of Public Health Research & Development. 2020;11(7):827-33.
12. Kiecolt-Glaser JK, Bennett JM, Andridge R, Peng J, Shapiro CL, Malarkey WB, et al. Yoga's impact on inflammation, mood, and fatigue in breast cancer survivors: a randomized controlled trial. Journal of Clinical oncology. 2014;32(10):1040-9.

13. Kim S, Ko YH, Song Y, Kang MJ, Lee H, Kim SH, et al. Pre-post analysis of a social capital-based exercise adherence intervention for breast cancer survivors with moderate fatigue: a randomized controlled trial. Supportive Care in Cancer. 2020;28(11):5281-9.
14. Littman AJ, Bertram LC, Ceballos R, Ulrich CM, Ramaprasad J, McGregor B, et al. Randomized controlled pilot trial of yoga in overweight and obese breast cancer survivors: effects on quality of life and anthropometric measures. Supportive Care in Cancer. 2012;20(2):267-77.
15. De Luca V, Minganti C, Borrione P, Grazioli E, Cerulli C, Guerra E, et al. Effects of concurrent aerobic and strength training on breast cancer survivors: a pilot study. Public health. 2016;136:126-32.
16. Milne HM, Wallman KE, Gordon S, Courneya KS. Effects of a combined aerobic and resistance exercise program in breast cancer survivors: a randomized controlled trial. Breast cancer research and treatment. 2008;108(2):279-88.
17. Moraes RF, Ferreira-Júnior JB, Marques VA, Vieira A, Lira CA, Campos MH, et al. Resistance training, fatigue, quality of life, anxiety in breast cancer survivors. The Journal of Strength & Conditioning Research. 2021;35(5):1350-6.
18. Nouri R, Braumann KM, ChamPiri BM, Schroeder J, Akochakian M. Cancer related fatigue and upper limb disabilities cannot improve after 6 weeks resistance training with Thera-Band in breast cancer survivors. International Journal of Applied Exercise Physiology. 2018;7(2):76-84.
19. Nyrop KA, Callahan LF, Cleveland RJ, Arbeeva LL, Hackney BS, Muss HB. Randomized controlled trial of a home-based walking program to reduce moderate to severe aromatase inhibitor-associated arthralgia in breast cancer survivors. The Oncologist. 2017;22(10):1238-49.
20. Ochi E, Tsuji K, Narisawa T, Shimizu Y, Kuchiba A, Suto A, et al. Cardiorespiratory fitness in breast cancer survivors: a randomised controlled trial of home-based smartphone supported high intensity interval training. BMJ supportive & palliative care. 2022;12(1):33-7.
21. Pagola I, Morales JS, Alejo LB, Barcelo O, Montil M, Olivan J, et al. Concurrent exercise interventions in breast cancer survivors with cancer-related fatigue. International journal of sports medicine. 2020;41(11):790-7.
22. Pinto B, Stein K, Dunsiger S. Peer mentorship to promote physical activity among cancer survivors: effects on quality of life. Psycho-Oncology. 2015;24(10):1295-302.
23. Qiao Y, Van Londen G, Brufsky JW, Poppenberg JT, Cohen RW, Boudreau RM, et al. Perceived physical fatigability improves after an exercise intervention among breast cancer survivors: a randomized clinical trial. Breast Cancer. 2022;29(1):30-7.
24. Rogers LQ, Fogleman A, Trammell R, Hopkins-Price P, Vicari S, Rao K, et al. Effects of a physical activity behavior change intervention on inflammation and related health outcomes in breast cancer survivors: pilot randomized trial. Integrative cancer therapies. 2013;12(4):323-35.
25. Rogers LQ, Vicari S, Trammell R, Hopkins-Price P, Fogleman A, Spenner A, et al. Biobehavioral factors mediate exercise effects on fatigue in breast cancer survivors. Medicine and science in sports and exercise. 2014;46(6):1077.

26. Saarto T, Penttinen HM, Sievänen H, Kellokumpu-Lehtinen P-L, Hakamies-Blomqvist L, Nikander R, et al. Effectiveness of a 12-month exercise program on physical performance and quality of life of breast cancer survivors. Anticancer research. 2012;32(9):3875-84.
27. Schad F, Rieser T, Becker S, Groß J, Matthes H, Oei SL, et al. Efficacy of tango argentino for cancer-associated fatigue and quality of life in breast cancer survivors: A randomized controlled trial. Cancers. 2023;15(11):2920.
28. Shobeiri F, Masoumi SZ, Nikravesh A, Moghadam RH, Karami M. The impact of aerobic exercise on quality of life in women with breast cancer: a randomized controlled trial. Journal of research in health sciences. 2016;16(3):127.
29. Soriano-Maldonado A, Diez-Fernandez DM, Esteban-Simon A, Rodriguez-Perez MA, Artes-Rodriguez E, Casimiro-Artes MA, et al. Effects of a 12-week supervised resistance training program, combined with home-based physical activity, on physical fitness and quality of life in female breast cancer survivors: the EFICAN randomized controlled trial. Journal of Cancer Survivorship. 2023;17(5):1371-85.
30. Stan DL, Croghan KA, Croghan IT, Jenkins SM, Sutherland SJ, Cheville AL, et al. Randomized pilot trial of yoga versus strengthening exercises in breast cancer survivors with cancer-related fatigue. Supportive Care in Cancer. 2016;24(9):4005-15.
31. Vardar Yağlı N, Şener G, Arıkan H, Sağlam M, İnal İnce D, Savcı S, et al. Do yoga and aerobic exercise training have impact on functional capacity, fatigue, peripheral muscle strength, and quality of life in breast cancer survivors? Integrative cancer therapies. 2015;14(2):125-32.
32. Yuen HK, Sword D. Home-based exercise to alleviate fatigue and improve functional capacity among breast cancer survivors. Journal of allied health. 2007;36(4):257E-75E.
33. Mawdsley D, Bennetts M, Dias S, Boucher M, Welton NJ. Model-based network meta-analysis: a framework for evidence synthesis of clinical trial data. CPT: pharmacometrics & systems pharmacology. 2016;5(8):393-401.
34. Dias S, Sutton AJ, Ades A, Welton NJ. Evidence synthesis for decision making 2: a generalized linear modeling framework for pairwise and network meta-analysis of randomized controlled trials. Medical Decision Making. 2013;33(5):607-17.
